# A novel solid-state, multi-layered biodegradable microbial inoculant system for rice straw composting: biocapsule design, characterization, and performance evaluation

**DOI:** 10.3389/fmicb.2026.1758888

**Published:** 2026-04-17

**Authors:** Uvin Eksith Senadheera, Dikkumburage Jasintha Jayasanka, Choolaka Hewawasam, Dhanushka Udayanga, Yuya Takimoto, Tadachika Nakayama

**Affiliations:** 1 Biosystems Technology Laboratory, Department of Biosystem Technology, Faculty of Technology, University of Sri Jayewardenepura, Homagama, Sri Lanka; 2 Faculty of Graduate Studies, University of Sri Jayewardenepura, Nugegoda, Sri Lanka; 3 Department of Civil and Environmental Technology, Faculty of Technology, University of Sri Jayewardenepura, Homagama, Sri Lanka; 4 Department of Applied Sciences, Faculty of Humanities and Sciences, Sri Lanka Institute of Information Technology, Malabe, Sri Lanka; 5 Department of Mechanical Engineering, Nagaoka University of Technology, Nagaoka, Japan; 6 Extreme Energy-Density Research Institute, Nagaoka University of Technology, Nagaoka, Japan

**Keywords:** bacterial encapsulation, biocapsule, biocompatibility, biodegradability, controlled release, hydroxyapatite nanoparticles, lignocellulolysis, rice straw composting

## Abstract

Lignocellulolytic microbial inoculants are widely used to enhance lignocellulosic waste composting, but their efficacy is often limited by environmental stress and uncontrolled release when conventional liquid inoculants are used. This study introduces a multi-layered biocapsule structure that sustains lignocellulolytic microbial activity and evaluates its composting efficiency through control experiments. A three-layered biodegradable biocapsule was designed using a rice straw biocomposite, humic acid, activated carbon, corn starch, carboxymethyl cellulose, and calcium alginate beads with encapsulated *Klebsiella–Enterobacter* consortium immobilized on hydroxyapatite nanoparticles. The biocapsule comprises a rice straw outer biocomposite shell in the outermost layer, a moisture retention hydrogel in the middle, and the encapsulated bacteria in calcium alginate beads in the core. The designed biocapsule was used in three treatments: intact biocapsule, powdered biocapsule, and a control (without inoculants), and the composting efficacy was evaluated against Sri Lankan Standards for compost products. Calcium alginate beads achieved 78.29% ± 9.57% and 84.45% ± 6.04% bacterial encapsulation efficiency and bacterial release, respectively, with heavy bacterial colonization in beads. The entire biocapsule reached 56.16% ± 1.65% biodegradation in 7 days. Intact biocapsule enhanced early lignocellulolysis, faster pH neutralization, and reduced electrical conductivity to 0.62 ± 0.00 while sustaining prolonged thermogenesis above 55 °C for 25 days. The intact biocapsule significantly improved compost nutrient availability, increasing total nitrogen, phosphorus, and potassium levels by 71.89%, 83.0%, and 60.66%, respectively, while achieving a total organic carbon loss of 53.30% and a Carbon: Nitrogen ratio decline of 72.83%.

## Graphical Abstract

**Figure:**
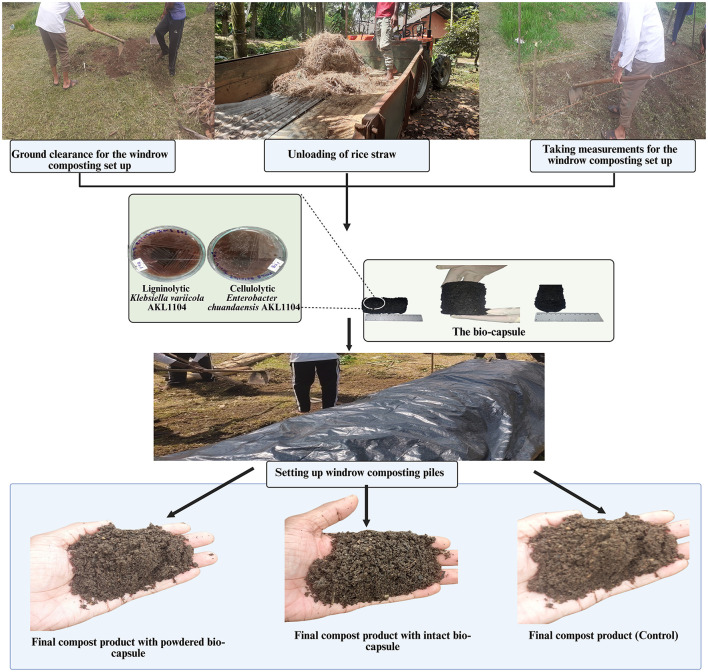

## 1 Introduction

Microorganisms are vital in lignocellulosic waste composting (Holman et al., 2016). Microbial activity causes biotransformation of plant nutrients, heat generation, and the depolymerization of larger compounds using hydrolytic enzyme activity. Indigenous microbes in agricultural waste naturally depolymerize the organic polymers; nevertheless, if the indigenous microbial population is not sufficiently diverse and gets adversely affected by environmental factors such as agricultural feedstock characteristics (e.g., secondary metabolite presence), composting can take an extended time and lower the composting efficiency (Greff et al., 2021).

According to Chi et al. (2020) and Gou et al. (2017), supplementing agricultural feedstock with beneficial microbes is an alternative strategy in composting that reduces the initial lag phase, accelerates organic waste decomposition, enhances humification, matures compost characteristics, and ultimately reduces costs (Ming et al., 2007). However, the use of beneficial microbes as external inoculants has been controversial for some time (López et al., 2002). Such debate on beneficial microbial inoculant efficacy should not be surprising, as the inoculant effectiveness is highly dependent on factors such as viability of inoculants after long-term storage, time of inoculation, competition among inoculant microbes and autochthonous microbes, conditions of composting, and feedstock characteristics (Wang J. et al., 2019).

According to Van Fan et al. (2017), beneficial microbial inoculants have a low efficiency, and issues with controlled release of microbes are observed in long-term full-scale composting. Furthermore, beneficial microbial inoculants with non-sporulating bacteria lose their viability during long-term storage. The inadequacy of attention during the formulation of liquid and solid microbial inoculants significantly reduces the cell viability and adaptability during release to the composting feedstock (Faure and Deschamps, 1991; Berninger et al., 2017; Lobo et al., 2018). Most microbial inoculants used for agricultural waste composting are liquid inoculants or utilize solid materials as microbial carriers (Zhou et al., 2015). However, liquid inoculants face a serious efficacy issue: cell viability and metabolic activity diminish rapidly after introduction into the composting feedstock.

One approach to circumvent the issue of cell viability and metabolic activity deterioration is the solid microbial inoculant formulations for composting. Among microbial inoculation delivery technologies, encapsulated microbial delivery technology is capable of circumventing the bottlenecks posed during conventional methods (Wu et al., 2010). Immobilization of beneficial microbial inoculants into biodegradable capsules via bacterial immobilization provides protection and resistance against competition from autochthonous microbes in agricultural composting feedstock, against detrimental physiochemical characteristics of the feedstock, and against predators until their biochemical processes initiate (Takei et al., 2008). Encapsulated beneficial microbe delivery releases bacteria in a controlled pattern, allowing them to colonize the feedstock. Calcium alginate (CA) has been widely used as a primary material for bacterial encapsulation due to its high biocompatibility and biodegradability. Therefore, CA can be used to control the release of beneficial bacterial cells into the composting feedstock. Furthermore, Prasad et al. (2017) propose the use of a low-cost gel carrier or gelling agent for moisture retention and improve the cell viability of microbial inoculants. Nevertheless, most solid-state microbial inoculants with sustained delivery, enhanced cell viability, and reduced toxicity innovations are surprisingly restricted to plant growth and soil quality improvements.

This study uses a fully biodegradable, thermally protected solid biocapsule containing lignocellulolytic bacteria harbored on hydroxyapatite (HAp) nanoparticles (HAp nanorod-assembled submicron particles) as a microbial inoculant delivery system to enhance rice straw (RS) composting. The bio-capsule comprises three layers: (1) outer RS-reinforced biocomposite for structural integrity of bio-capsule; (2) Humic acid-supplemented carboxymethyl cellulose for temperature fluctuation protection and moisture retention; (3) HA microparticle-loaded CA capsules with the lignocellulolytic bacterial consortium. The objective of this article is to evaluate the efficiency of solid-state microbial inoculants on RS compost and to assess the survival of lignocellulolytic bacterial inoculants against temperature fluctuations (hot and cold), moisture reduction, and long-term storage under the protection of a designed bio-capsule. The results of this study can serve as a baseline for the development of sustained-release solid-state beneficial bacterial inoculants for composting agricultural waste and for comparing their success against conventional liquid inoculants.

## 2 Materials and methods

### 2.1 Formulation of biocapsule raw materials

#### 2.1.1 Synthesis of HA nanoparticles (HAp nanorod-assembled submicron particle)

Twelve mM diammonium phosphate [(NH_4_)_2_HPO_4_] solution (60.0 mL) was prepared, and the pH was adjusted to pH 6.0 ± 0.1 with 1.0 M HNO_3_ while shaking at 160 RPM. Under vigorous mixing conditions (800 RPM, baffled 250 mL beaker, four-blade PTFE impeller), 40 mL of 20 mM calcium nitrate tetrahydrate [Ca (NO_3_)_2_·4H_2_O] was introduced to the solution for 120 s through a dip-tube at a depth of ~5 mm below the liquid surface while pH was maintained near pH 5.00 with 1.0 M HNO_3_. The solution was homogenized at 800 RPM for 2 min. 0.9 g of anhydrous sodium citrate (Na3C6H5O7) was introduced and stirred for 10 min to ensure complete dissolution. Eighty mL of the solution was transferred into a 100 mL Teflon bottle positioned in a stainless-steel autoclave and sealed, and ultimately heated to 180 °C for 2 h. The vessel was allowed to reach room temperature. The precipitates cooled down to room temperature were washed in distilled water for 3 times, centrifuged (12,000 RPM, 30 min at 25 °C), and freeze-dried overnight (Jarudilokkul et al., 2006).

### 2.2 Synthesis of HuA from RS

**Figure 1:**
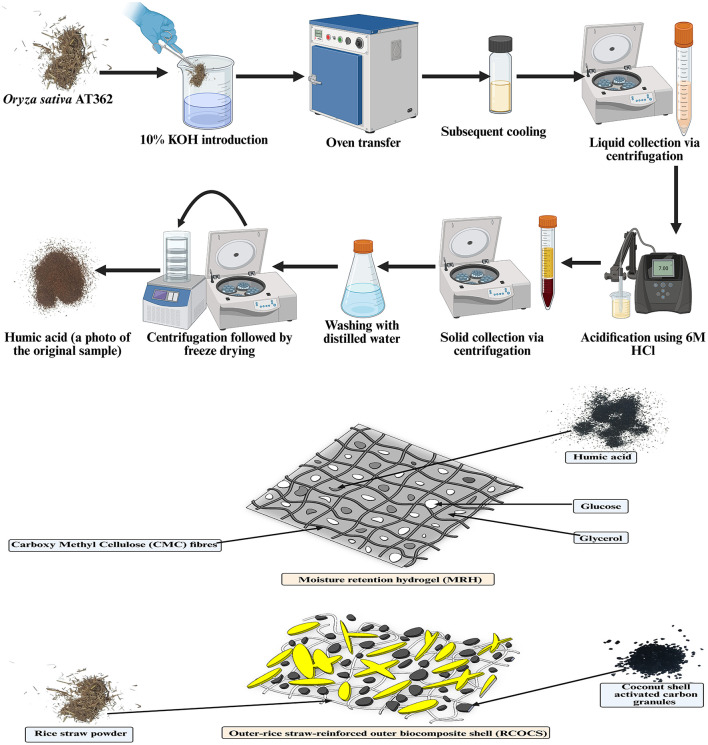
A schematic representation of humic acid production for the biocapsule **(Upper)**. A schematic representation of the structure of the moisture retention hydrogel (MRH) and outer rice straw reinforced biocapsule composite shell (RSOCS) **(Lower)**.

1.20 g of RS (*Oryza sativa* AT362) (sieved through mesh sieve size of 0.149 mm) mixed in 10% KOH solution in a reaction kettle (100 mL) with polytetrafluoroethylene liner. The kettle was sealed and heated to 200 °C for 24 h in an oven. After the reaction mixture reaches room temperature, the content was centrifuged at 5, 000 RPM for 10 min to separate insoluble particles and obtain crude HuA. The solution pH was adjusted to 1.0 with 6 M HCl, and the mixture was centrifuged at 5,000 RPM for 10 min to obtain crude HuA. The crude HuA was cleaned three times with distilled water and freeze-dried overnight to obtain the final HuA. The average yield of HuA was calculated using Equation 1 (Wang et al., 2022). The schematic pathway for the HuA production is shown in Figure 1.


$$\begin{array}{l} HuA\;\left(\%\right)=\frac{m_{HuA}}{m_{RS}}\times 100 \end{array}$$ (1)

Where *m*_*HuA*_,*m*_*RS*_ are the dry weights of final HuA and RS, respectively.

### 2.3 AC (activated carbon) synthesis from coconut shells

Hundred gram of washed, dried, and pulverized coconut shells (CS) were heated in a furnace at 600 °C for 2 h. The carbonized CS was ground and sieved to a 1–2 mm particle size to improve the homogenization of the CS with steam during the activation process (Yagmur and Kaya, 2021; Jedynak and Charmas, 2024). The carbon yield (*C*_*carbonised*_) was calculated using Equation 2 in triplicate (*n* = 3) measurements. The carbonized CS was further cooled down to room temperature in an air-tight desiccator.


$$\begin{array}{l} C_{carbonised}\;yield\;\left(\%\right)=\left(\frac{m_{carbonised}}{m_{pre-treated}}\right)\times 100 \end{array}$$ (2)

Where *m*_*carbonised*_, *m*_*pre*−*treated*_ are the weights of the sample after carbonization and pre-treated CS, respectively, in grams. The carbonized CS was introduced into a stainless-steel reactor heated to 300 °C by an electrical tube furnace for 30 min, and the heat was subsequently increased to 800 °C for 5 h. Steam injection to the pyrolyzed CS was maintained at 120 mL h^−1^ into the reactor for activation of the pyrolyzed CS. The reactor was cooled to room temperature after activation. The AC was thoroughly washed five times using distilled water. The washed AC was dried at 110 °C for 2 h in an oven, cooled to room temperature, and stored in a desiccator (Hidayu and Muda, 2016). ImageJ software was used to analyze the pore-size distribution in the prepared AC.

### 2.4 Characterization of HA, HuA, and AC

Ten mg of freeze-dried sample was used for FTIR (Fourier Transform Infrared Spectroscopy), SEM (Scanning Electron Microscopy) analysis using the protocol described by Dar et al. (2018) using for SEM analysis, Carl Zeiss™ EVO 18 Research instrument. 10mg of the samples were taken and mixed with 1,000 mg of KBr (spectroscopic grade) powder in an agate mortar, then pressed into discs (10,000 psi). The FTIR spectra were recorded in a Thermo Scientific Nicolet iS10 FT-IR spectrometer™ (Thermo Electron Scientific Instruments LLC, Waltham, Massachusetts, United States). To analyze the stoichiometry of Ca/P ratio of the synthesized HA, SEM-EDAX was used (protocol as same as in Section 2.6.1).

### 2.5 Production of the capsule counterparts

#### 2.5.1 Outer-RS-reinforced outer composite shell (RSOCS)

The dry RS is dried at 45 °C for 6 h in an oven and then cut into 2–3 mm pieces. The oven-dried RS (*Oryza sativa* AT362) is then freeze-dried overnight to remove any residual moisture. The cut pieces are then shredded for 9 min into finer pieces (0.8−1.2 mm long). The prepared RS fibers were autoclaved twice by placing them in an autoclavable 500 mL beaker and covering them with aluminum foil. The prepared RS fibers (13% (w/v) and AC are mixed with 95 mL of distilled water and corn starch 30% (w/v), followed by the addition of 8% (w/v) AC with constant mixing until the composite materials are thoroughly mixed. All the materials are autoclaved twice before making the bio-composite.

#### 2.5.2 Moisture-retention hydrogel (MRH)

Thirty-five g of CMC (carboxy methyl cellulose) is mixed with 150 mL of distilled water in batches. First, 5 g of CMC is mixed with 150 mL of distilled water, followed by another batch of CMC, until a total of 35 g of CMC is mixed with distilled water (7 times). Mixing is performed in a vortex mixer at 40 °C. Thirty mL of glycerol is mixed with the CMC/distilled water mixture in batches of 6 mL in a vortex mixer at 40 °C. 0.25 g of HuA is added in batches of 0.05 g to the CMC/distilled water/glycerol mixture. Finally, 1.65 g of glucose is mixed with the CMC/HuA/distilled water/glycerol mixture and thoroughly mixed for 45 min continually with a spatula (all ingredients are mixed by hand with intermittent stops of the vortex mixer at 10 min intervals). After the slurry turns off-white and becomes non-sticky, it is placed in a 250 mL beaker, and the mouth of the beaker is sealed off with aluminum foil. The slurry is then autoclaved twice. A schematic representation of MRH and RSOCS is shown in Figure 1.

#### 2.5.3 Encapsulation of the lignocellulolytic bacterial cells in CA beads

**Figure 2:**
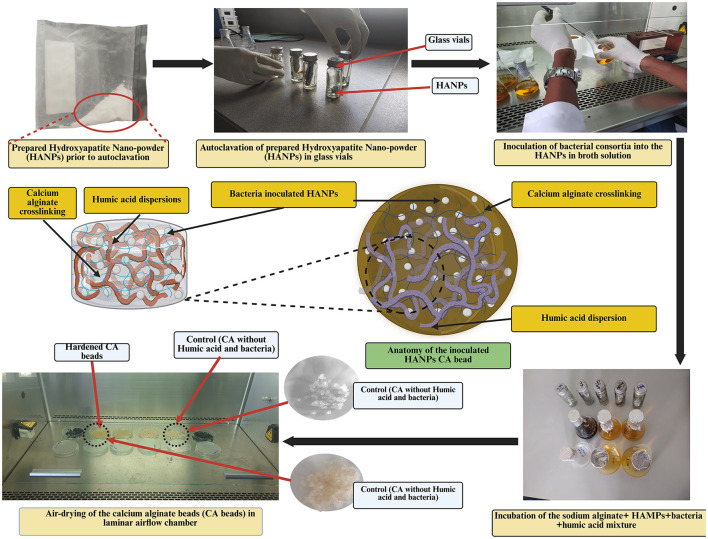
The workflow for the bacterial encapsulation in calcium alginate beads.

Previously isolated cellulolytic *Enterobacter chuandaensis* (Accession code: PP989911) and ligninolytic *Klebsiella variicola* (Accession code: PP989916) RS bacterial endophytes were used for CA encapsulation by the authors (Senadheera et al., 2025). The endophytic isolates were co-cultivated (1:1) in NA broth (tryptone, 10.0 gL^−1^; beef extract, 3.0 gL^−1^; NaCl, 5.0 gL^−1^) at 37 °C and 200 RPM until the population reached 1 × 10^6^*CFU mL*^−1^. Prepared HA microparticles (0.25 g), 50.0 mL of distilled water (sodium alginate), SA (3.0%), and HuA (0.5% w/v) were mixed in NA broth (tryptone, 10.0 gL^−1^; beef extract, 3.0 gL^−1^; NaCl, 5.0 gL^−1^) and autoclaved twice. The bacterial suspension was aseptically added to the previously autoclaved mixture with HA microparticles and incubated in a shaker at 37 °C and 120 RPM for 24 h. The mixture containing inoculated bacteria, HA microparticles, and SA was injected into an autoclaved 0.5 M CaCl_2_ solution using a 5.0 mL injection needle. Formed CA beads were removed from the CaCl_2_ solution after 30 min and washed three times with distilled water and dried in the laminar-air flow for 35 min. Figure 2 shows the workflow for bacterial encapsulation.

### 2.6 Characterization of the biocapsule components

#### 2.6.1 Physicochemical and morphological characterization of RSOCS, MRH, and CA beads

SEM, SEM-EDAX, FTIR, and thermogravimetric characterization were conducted by the method described by Atta et al. (2022) for characterization of RSOCS, MRH, and CA beads. Freeze-dried samples for overnight were analyzed using ATR (Attenuated Total Reflection) FTIR using Smar tiTX ATR accessory of Thermo Scientific Nicolet iS10 FT-IR spectrometer™. For SEM and EDAX analysis, the Carl Zeiss™ EVO 18 Research instrument was used. TGA analysis was conducted using an SDT Q600 (V20.9 Build 20) (TA Instruments (a subsidiary of Waters Corporation), New Castle, United States) instrument with a DSC-TGA standard module. Fifteen mg of the freshly prepared samples were weighed on an alumina pan and subjected to the ramp methodology at $10C\overset{-1}{\min}$ heating rate and the temperature was increased from room temperature to 600.00 °C. The analysis was performed under *N*_2_ atmosphere with a purge flow of $100\;mL\;\overset{-1}{\min}$ with a sampling interval of 0.5s per point. The moisture retention analysis of MRH and the solubility of RSOCS and MRH were measured following the protocol described by Ojagh et al. (2010). Equation 3 was used for moisture retention analysis of MRH (∅) Equation 4 was used for solubility analysis of RSCOS and MRH (∂), respectively (*n* = 3).


$$\begin{array}{l} \emptyset =\frac{\left(m_{dried}-m_{immersed}\right)}{m_{dried}\times 100} \end{array}$$ (3)

$$\begin{array}{l} \partial =\frac{\left(m_{\;before\;drying}-m_{after\;drying}\right)}{m_{before\;drying}\times 100} \end{array}$$ (4)

In Equation 3, *m*_*dried*_,*m*_*immersed*_ are the dry weights after immersion in water and drying, respectively, and in Equation 4, *m*_*before drying*_, *m*_*after drying*_ are the hydrogel weights before drying and after drying, respectively. The capsule size of 100 randomly selected CA beads (diameter) was measured using 150 mm Vernier calipers with triplicate measurements. SEM and SEM-EDAX for CA beads were performed according to the protocol by Tran et al. (2024). For CA beads, FTIR analysis was performed as mentioned in Section 2.6. The swelling analysis was carried out in physiological saline (0.85% w/v) at room temperature in bead diameter expansion (Diameter expansion coefficient; DEC) and gravimetric methods (Weight expansion coefficient;WEC) using Equations 5, 6 (Chen et al., 2008; Sanli and Solak, 2009) (*n* = 100).


$$\begin{array}{l} DEC=\frac{D_{swollen}}{D_{dried}} \end{array}$$ (5)

$$\begin{array}{l} WEC=\frac{m_{swollen}}{m_{dried}} \end{array}$$ (6)

*D*_*swollen*_, *D*_*dried*_ are swollen and dried CA average bead diameter; *m*_*swollen*_, *m*_*dried*_ are swollen and dried, with average weights of CA beads, respectively. The number of unencapsulated bacteria in CA beads was calculated using the plate count method and bacterial encapsulation efficiency (η) is calculated as follows in Equation 7 (*n* = 100).


$$\begin{array}{l} \eta =\frac{\left(N_{initial}-N_{unencapsulated}\right)}{N_{initial}}\times 100 \end{array}$$ (7)

*N*_*initial*_, *N*_*unencapsulated*_ are initial and unencapsulated CFUs in 0.5 M CaCl_2_, respectively. The release rate (ζ) was calculated by immersion of CA beads in physiological saline for 14 d in room temperature. ζ is given by Equation 8 (*n* = 100).


$$\begin{array}{l} \zeta =\frac{\left(N_{unencapsulated}\right)}{N_{initial}}\times 100 \end{array}$$ (8)

The sphericity (σ) of CA beads was calculated as described by Tran et al. (2024) using Equation 9 (*n* = 100).


$$\begin{array}{l} \sigma =\frac{\left(d_{maximum}-d_{miniumum}\right)}{\left(d_{maximum}+d_{miniumum}\right)} \end{array}$$ (9)

#### 2.6.2 Kinetics of bacterial release

Cumulative load of released and viable bacteria in different time intervals (*M*_*t*_) was analyzed by the colony count method. For a deeper insight into bacterial cell release from entrapment, optimized swellable encapsulated systems have been used (Lamprecht et al., 2003; Roy et al., 2008; Wu and Liu, 2007). According to Wu et al. (2010), number of studies are used for explaining the cell release mechanisms from swellable spheres, no single mathematical models can predict all of the experimental observations. The bacterial cell release was analyzed using the empirical Equation 10 proposed by Lamprecht et al. (2003), Roy et al. (2008), and Wu and Liu (2007).


$$\begin{array}{l} \frac{M_{t}}{M_{\infty }}=Kt^{n} \end{array}$$ (10)

Where $\frac{M_{t}}{M_{\infty }}$ is the fraction of the released bacterial cells at the time *t*; *K* is a constant related to the geometric and structural characteristics of the gel be *n* is a swelling component, which indicates the bacterial cell release mechanism. The calculations of *K* and *n* were analyzed up to 40% release of the bacterial cells. The diffusional exponent *n* specifically gives insights into the mechanism of bacterial cell release. For spheres, the values of *n* of 0.43–0.85 and indicative of both diffusion-governed bacterial cell release and controlled bacterial cell release. According to Ritger and Peppas (1987) and Siepmann and Peppas (2012). The values of *n*>0.85 are case-II bacterial cell release mechanisms linked to alginate polymer relaxation during gel bead swelling. The diffusion coefficient for the bacterial cell release was calculated according to the Equation 11 given below. All the bacterial release rate readings were taken in triplicate (*n* = 3).


$$\begin{array}{l} D={\left(\frac{k}{{\left(4\pi^{2}\right)}^{n}}\right)}^{\frac{1}{n}} \end{array}$$ (11)

The bacterial cell release mechanism of the alginate beads can be adequately described according to Peppas model, as the model is based on a phenomenological perspective, and this is due to the model's ability to describe swelling devices (Taresco et al., 2022). The regression models and the changes in $\frac{M_{t}}{M_{\infty }}$ for the total bacterial release, cellulolytic and ligninolytic bacterial release are shown graphically. For all types of cell release mechanisms, the correlation coefficient (*R*^2^) was above 0.91, and the cell release data fitted adequately with statistical significance (Roy et al., 2008; Singh et al., 2008; Wu and Liu, 2007; Yang et al., 2010).

### 2.7 Biodegradability, biocompatibility, and interlayer compatibility of individual biocapsule components

The protocol described by Van Wylick et al. (2022) was used for biodegradability studies. Phytotoxicity of the degradation product is analyzed by the protocol described by Enaime et al. (2020) using Mung seeds [*Vigna radiata* (L.) R. Wilczek]. The relative seed germination (ψ), relative root growth (λ) and phytotoxicity index (μ), germination index (*GI*) are given by Equations 12–15, respectively (*n* = 3).


$$\begin{array}{l} \psi =\left(\frac{GS_{sample}}{GS_{control}}\right)\times 100 \end{array}$$ (12)

$$\begin{array}{l} \lambda =\left(\frac{RL_{sample}}{RL_{control}}\right)\times 100 \end{array}$$ (13)

$$\begin{array}{l} \mu =1-\left(\frac{RL_{sample}}{RL_{control}}\right) \end{array}$$ (14)

$$\begin{array}{l} GI=\frac{\psi \times \lambda }{100} \end{array}$$ (15)

The interlayer biocompatibility of the capsule was evaluated using SEM and FTIR (same protocol as in Section 2.6.1). Samples were obtained from interlayer junctions (RSOCS-MRH junction, CA-MRH junction, and RSOCS-CA junction) at three different locations and mixed using the quartering technique to obtain a representative sample.

### 2.8 Assembly of the biocapsule

#### 2.8.1 Loading of bacterial encapsulated CA beads and assembly of the biocapsule

The RSOCS is synthesized according to the protocol mentioned in Section 3.1. RSOCS holds the structural integrity of the biocapsule. The RSOCS is made in two steps. First, the cylindrical shell is made, and thereafter, the lid is molded to enclose the CA beads. For the molding of the cylindrical shell, the RSOCS is poured into the surface-sterilized aluminum hollow mold with space between the inner and outer layers. The innermost diameter of the mold is 4 cm. The outermost diameter of the mold is 5.8 cm. The outermost height of the mold is 12.4 cm. The RSOCS slurry (100 g) is filled into the space between the inner and outer walls (0.5 cm inner space) and subjected to two-stage drying with primary drying at 45 °C for 9 h, followed by secondary drying at 65 °C for 10 h, and allowed to cool to room temperature. The cross-sectional thickness of the mold is 0.2 cm. The biocomposite shell is then UV-sterilized under a laminar airflow hood for 45 min. The outer wall of the biocomposite shell is covered with 3 layers of aluminum foil. The interior wall of the biocomposite shell is coated with MRH, three times using the dipping method described by Fernández et al. (2010) until the MRH dipping thickness becomes 0.2 cm in average (measured using vernier caliper using after averaging the thickness from *n* = 10 places. The MRH-coated RSCOCS shell is dried for 45 min in a laminar airflow under UV light. The bacteria-encapsulated CA beads are placed inside the biocomposite shell using a sterilized funnel in a laminar airflow. The RSOCS lid is molded with the aluminum mold. The aluminum mold used for this has a diameter of 5.4 cm with a thickness of 0.2 cm. The RSOCS lid has a diameter of 2.5 cm and a thickness of 0.5 mm. The lid is also subjected to the procedures mentioned for the RSOCS cylindrical shell. The one cylindrical face of the lid is covered with 3 layers of aluminum foil, and the other circular face of the lid is coated with MRH as mentioned above until the thickness reaches 0.2 cm on average (measured using a vernier caliper after averaging the thickness from *n* = 10 places). The biocapsule is standardized using CFUs per one CA bead (*n*_*b*_). The standardization was as follows. 0.1 g of encapsulated beads was completely dissolved in 9.9 mL of physiological saline (0.85% w/v) with vigorous shaking at 120 RPM. The released bacteria were enumerated using the plate count method (*n* = 100). The total bacterial count was taken using LB (Luria Bertani) agar and the cellulolytic count and ligninolytic cell count was taken by plating in cellulolytic (2.0 gL^−1^ (NH_4_)_2_SO_4_; 0.5 gL^−1^ MgSO_4_; 1.0 gL^−1^ K_2_HPO_4_; 0.5 gL^−1^ NaCl; 5.0 gL^−1^ alkaline lignin; 20.0 gL^−1^ agar powder; 1.0 L H_2_O) and ligninolytic (1 gL^−1^ KH_2_PO_4_; 0.5 gL^−1^ K_2_SO_4_; 0,5 gL^−1^ NaCl; 0.01 gL^−1^ FeSO_4_, 0.01 gL^−1^ MnSO_4_, 1 gL^−1^ NH_4_NO_3_, 10 gL_−1_ CMC, 20 gL^−1^ agar powder and 1.0 L H_2_O) selective media. Supplementary Figure 1 shows the standardization protocol and capsule dimensions.

Enumeration leads to 0.94 ± 0.05 × 10^6^*CFU* per bead of total bacteria, and the cellulolytic and ligninolytic cell counts were 0.43 ± 0.04 × 10^6^*CFU*and 0.43 ± 0.03 × 10^6^*CFU* per bead, respectively. To determine the number of encapsulated CA beads to be included in the biocapsule treatments (*n*_0_), the following strategy was used.

The average bacterial cell counts of the starting feedstock (*CFUmL*−1 *control*) materials after mixing were calculated using the plate count method (*n* = 3). Thereafter, the following Equation 16 was used to establish a relationship between the number of bacteria encapsulated in CA beads wanted and the starting bacterial population of the *T*_*control*_.


$$\begin{array}{l} {CFUmL^{-1}}_{control}=n_{0}\times \;n_{b} \end{array}$$ (16)

**Figure 3:**
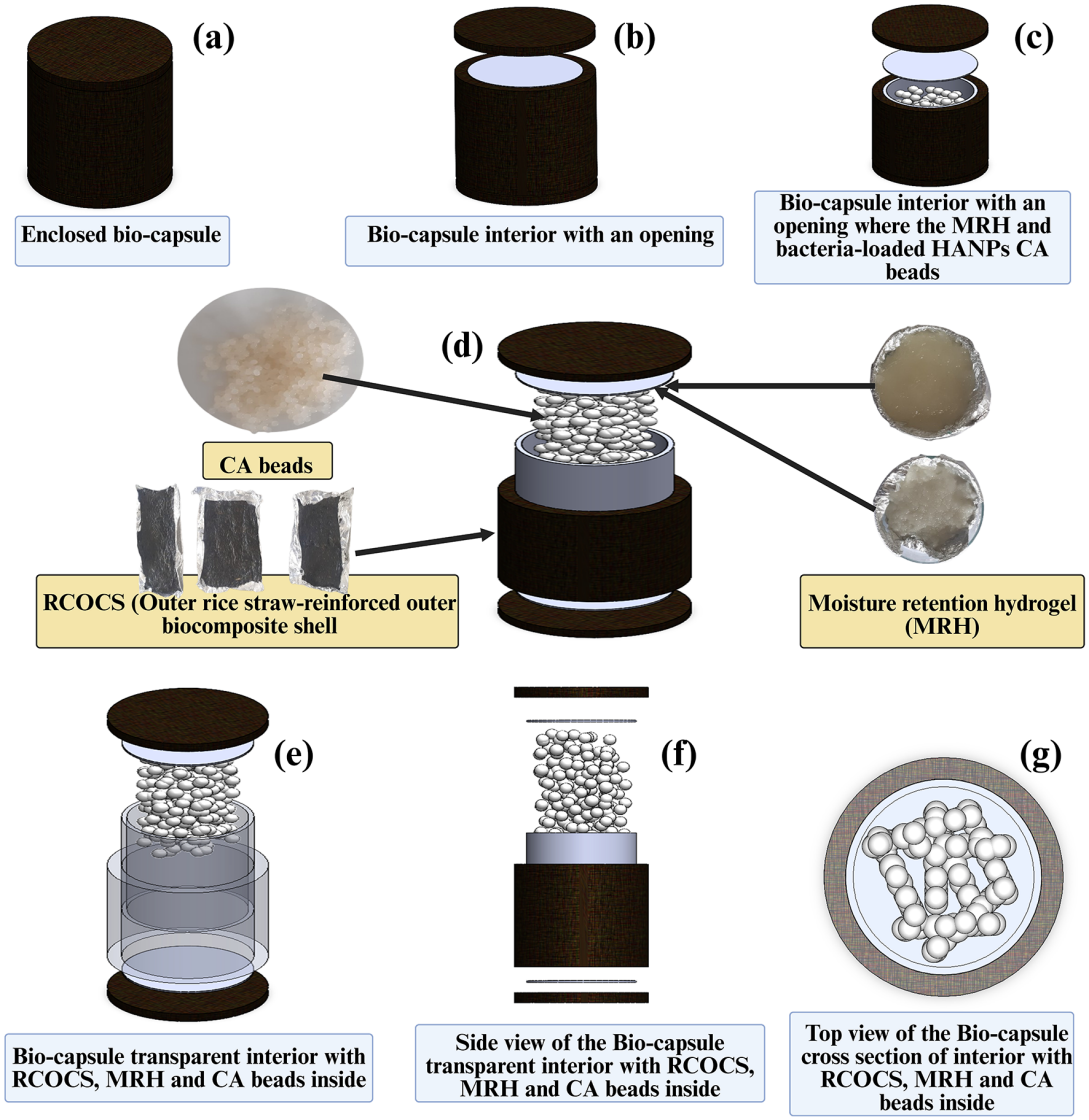
Anatomy of the biocapsule. **(a)** Side view of the biocapsule; **(b)** Side view of the biocapsule where the RCOCS lid is open, the MRH interior can be seen; **(c)** Slightly elevated side view of the biocapsule where the RCOCS lid and MRH is lifted to reveal the CA beads with bacteria; **(d)** On the left: Bacteria-encapsulated calcium alginate beads (top), RCOCS sheets (bottom), In the middle: slightly elevated expanded side view of the biocapsule where RCOCS layer, MRH layer and calcium alginate beads are visible, On the right: MRH on petri plates; **(e)** Slightly elevated, expanded and transparent side view of the biocapsule with RCOCS, MRH layers and CA beads, **(f)** Expanded side view of the biocapsule with RCOCS, MRH layers and CA beads; **(g)** Top view of the biocapsule cross section.

The ${CFUmL^{-1}}_{control}$ is 2.64 × 10^9^*CFUmL*^−1^ and the average *n*_*b*_ is 0.94 ± 0.05 × 10^6^*CFUmL*^−1^ in CA beads. Therefore, the *n*_0_ is ~28.08 CA beads per biocapsule, and it was rounded off to 28 beads per capsule. The RSCOCS cylindrical shell is closed with a sterilized RSCOCS lid. The top lid is connected to the capsule by applying MRH slurry using a surface-sterilized glass rod. Figure 3 shows the anatomy of the biocapsule.

### 2.9 Biodegradability and biocompatibility of the biocapsule

The biodegradability and biocompatibility of the capsule are evaluated as mentioned in Section 2.7.

### 2.10 Composting effect testing of biocapsule

RS was prepared following the protocol described by Zakarya et al. (2018). Cow dung was obtained from lactating dairy cows at the District Agriculture Training Center, *Gabadawatte, Homagama, Sri Lanka*. The composting analysis will be performed in a Completely Randomized Design (CRD) with three experimental setups. The pile composition is given in Table 1. Composting is carried out using the outdoor windrow method. Raw materials are well-mixed and arranged into long strips (20 *m*; *L*×2 *m*; *W*×1.5 *m*; *H*). The temperature profile was analyzed as defined by Du et al. (2021). The initial moisture content of all feedstock materials was maintained at 60% by adding water as required. Sampling was conducted from 0 d to 60 at 5 d intervals from four random places within the pile, and a representative sample (1,000 mg) was obtained by the quartering method. The sample was air-dried, ground, and sieved through a 2 mm sieve. pH, electric conductivity (EC) (*dsm*^−1^), moisture content by dry mass (*MC*_*dm*_%), total *N* by dry mass (*TN*_*dm*_%), total *P* content (P_2_O_5_) by dry mass (*TP*_*dm*_%), total *K* content K_2_O) by dry mass (*TK*_*dm*_%), total organic C by dry mass (*TOC*_*dm*_%), $\frac{C}{N}$ ratio and sand content (*S%*) is analyzed according to the Sri Lankan Standards (SLS) 1636: 2019 for compost made from raw materials of agricultural and animal origin (Sri Lanka Standards, 2023). All composting tests were done in triplicates (*n* = 3).

**Table 1:** The pile contents for treatments in the composting process.

Treatment	Composting pile content
*T*_*control*_	Rice straw^a^ + cow dung^b^ (control)
$T_{biocapsule}^{S}$	Rice straw^a^ + cow dung^b^ + solid biocapsule (intact)
$T_{biocapsule}^{PS}$	Rice straw^a^ + cow dung^b^ + solid biocapsule (powdered)^c^

^a^Rice straw 80 kg.

^b^Cow dung 288 kg. *T*_*control*_ treatment has no bacterial inoculum used, $T_{biocapsule}^{S}$*;* Solid or intact capsule, $T_{biocapsule}^{PS}$; break open and powdered capsule.

^c^The $T_{biocapsule}^{PS}$ was prepared using a centrifugal ball mill (model PM100) where a cylindrical steel jar with a volume of 50*cm*^3^) with five 10 mm steel balls and the milling was conducted for 30 s at 600 RPM (Ambrosio-Martín et al., 2014).

### 2.11 Statistical analysis

Data were analyzed using one-way ANOVA, followed by Tukey's HSD (Honestly Significant Difference) for pairwise comparisons. Normality and homogeneity of variance were verified before parametric tests; non-parametric alternatives (Kruskal–Wallis, Mann–Whitney) were applied, where assumptions were violated. All results are expressed as mean ± SD (standard deviation). A measurement was considered significant at α = 0.05. Significant and highly significant differences were considered significant at *p* < 0.01 and *p* < 0.001. Interaction effects and effect sizes (η) were calculated where applicable.

## 3 Results

### 3.1 Characterization of the biocapsule raw materials and individual biocapsule components

**Figure 4:**
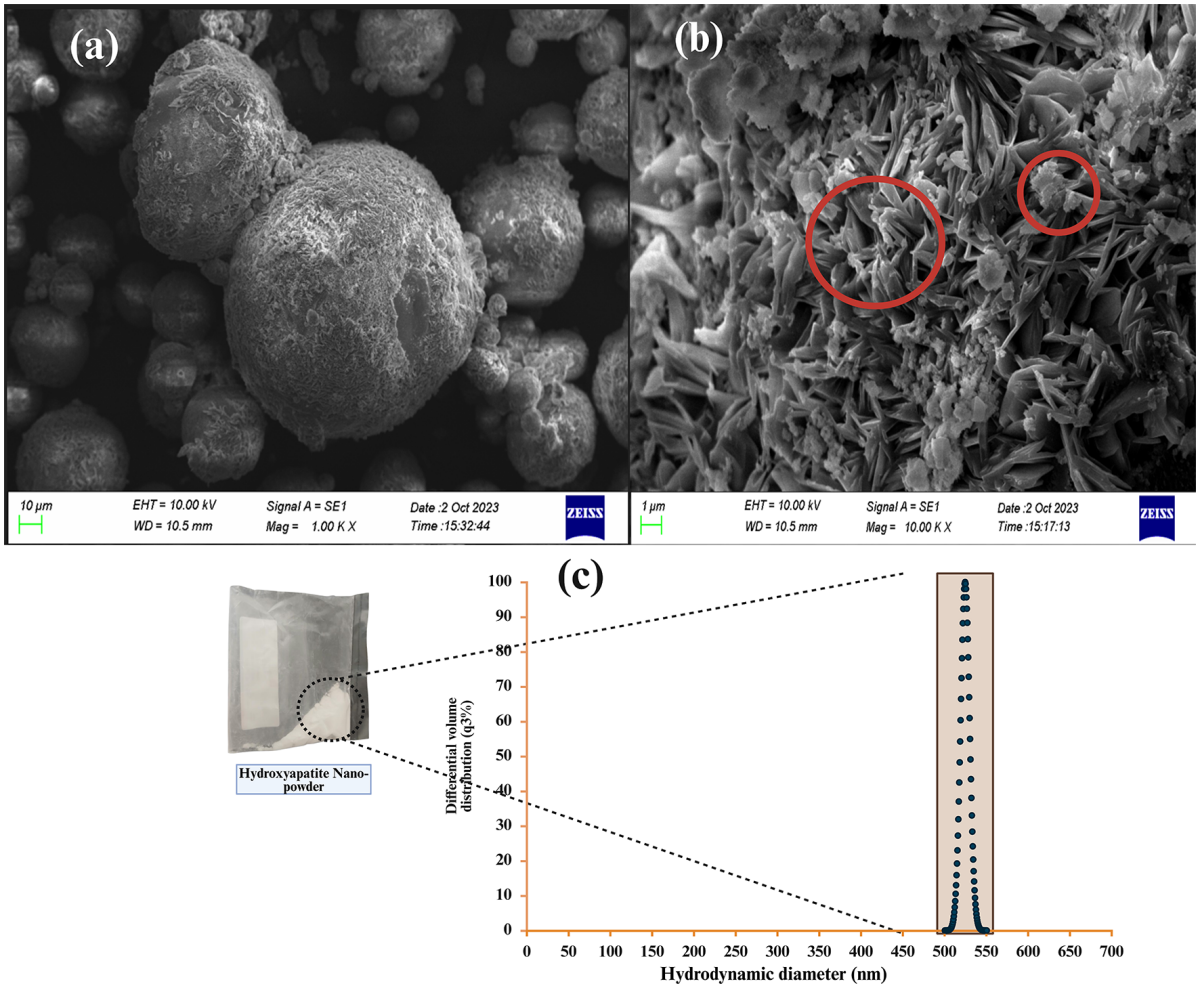
**(a)** The SEM micrograph of the surface of HAp nanorod-assembled submicron particles; **(b)** The plate-like regions with distinct boundaries between packed nanorods; **(c)** The particle size distribution of HAp nanorod-assembled submicron particles.

**Figure 5:**
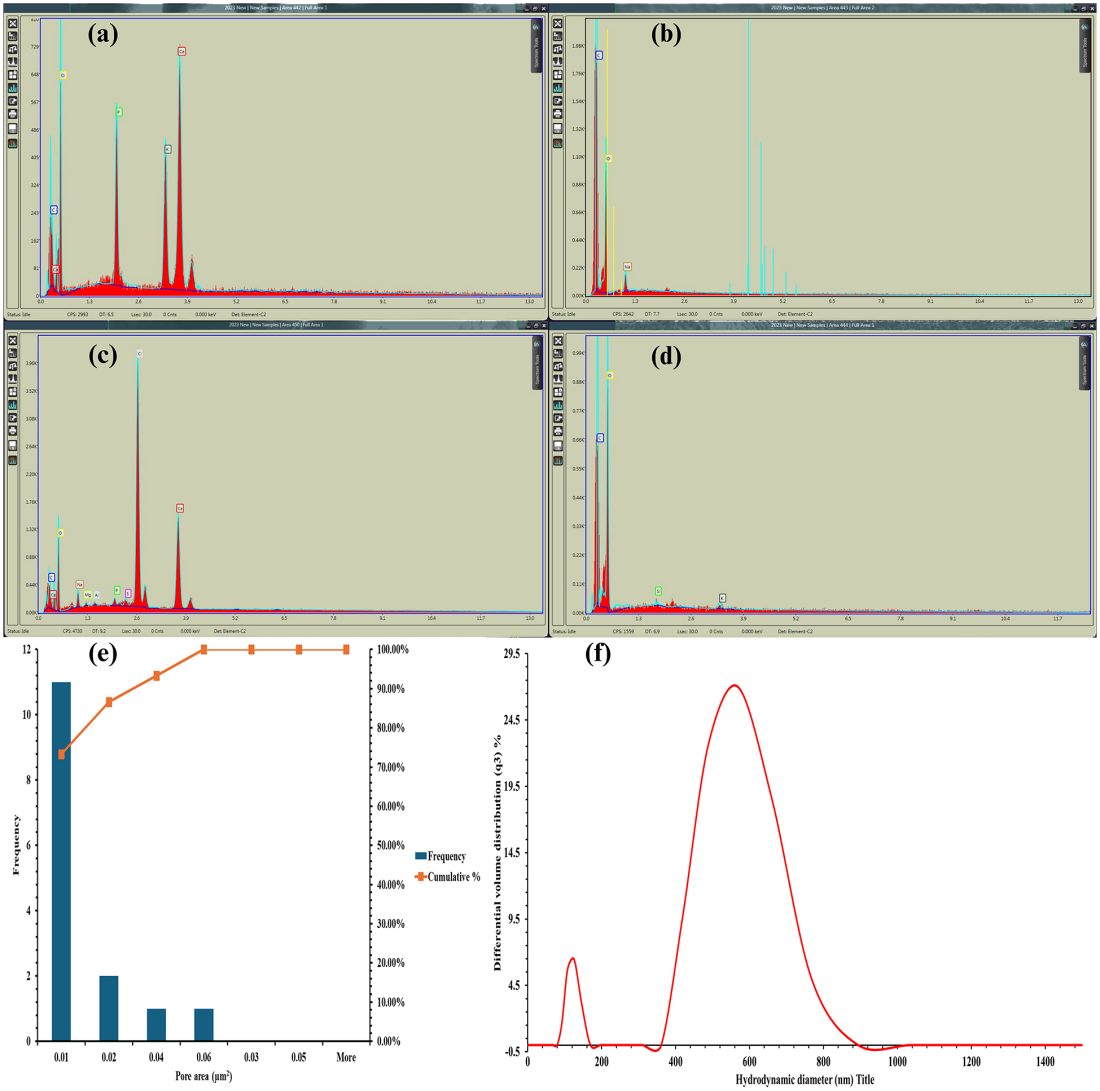
**(a)** SEM-EDAX of HAp nanorod-assembled submicron particle; **(b)** SEM-EDAX of Moisture Retention Hydrogel; **(c)** SEM-EDAX of calcium alginate beads; **(d)** SEM-EDAX of outer-RS-reinforced outer composite shell; **(e)** pore size distribution of activated carbon; **(f)** particle size distribution of the bacteria adhered HAp nanorod-assembled submicron particles.

**Figure 6:**
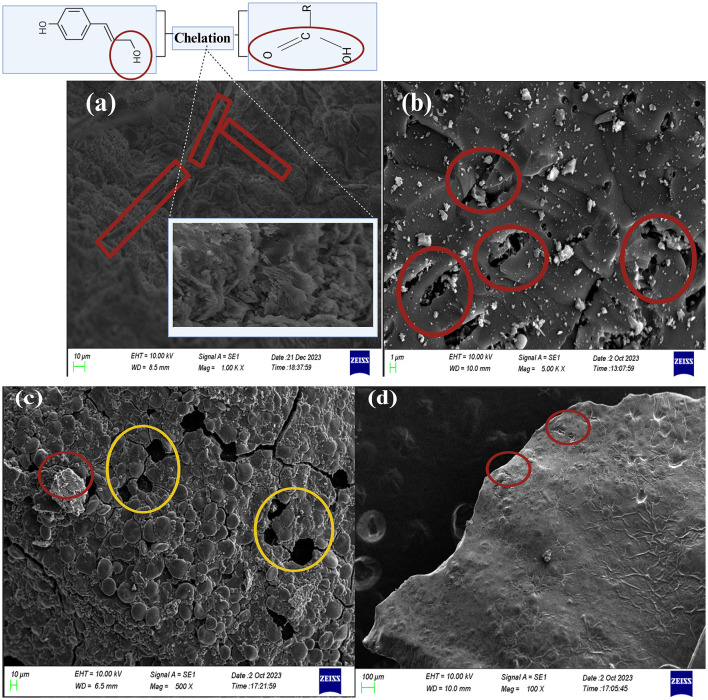
**(a)** SEM micrograph of humic acid with thin thread-like structures are visible which might grow into larger sheets and rings, highlighted in bold red, **(b)** SEM micrograph of activated carbon with dissolution of lignin and other mineral compounds during activation, which ultimately causes micro and mesopore formation (Tu et al., 2019), **(c)** SEM micrograph of outer rice straw reinforced biocapsule composite shell with pores visible and the dispersion of activated carbon (highlighted), **(d)** SEM micrograph of moisture retention hydrogel with dense packing and occasional cracks on the surface (highlighted).

The SEM images of the HAp nanorod-assembled submicron particle are shown in Figure 4. The HAp particles possess an average size of 525 nm for HAp nanorod-assembled submicron particles (Figure 4c). EDAX spectra of HAp nanorod-assembled submicron particles exhibited the peaks from C, O, Ca, and P, indicating that the signals originate from the constituent elements of HAp precursors (Figure 5a). The C and O signals emitted by all the biocapsule components originated from the polymeric C in them (Figures 5a–d). The Ca signal in CA beads is due to the formation of calcium alginate during the calcination of sodium alginate and calcium alginate (Figure 5c). The P signal in Ca beads is due to the presence of HAp nanorod-assembled submicron particles entrapped in the bead (Figure 5c). The weak Si peak on RSOCS is due to the silica deposits on rice straw fibrils on RSOCS (Figure 5d) (Akhtar et al., 2023). The pore sizes of AC in RSOCS were highly homogeneous, and 73.33% of the total pores were within the ~0.01μ*m*^2^. The average pore area was 0.016 ± 0.014 μm^2^. This high homogeneity shows that the steam activation process allows the distribution of pores with uniform sizes (Figure 5e). The HuA yield from RS was 16.6 ± 0.003%. SEM images of HuA and AC are shown in Figures 6a, b, respectively. The purity index for classification and the humic quality of the extracted HuA were analyzed by the protocol described by Fuentes et al. (2016) using the following Equation 17.


$$\begin{array}{l} S_{G}=\alpha G\times R_{\frac{1720}{1620}}+\beta G\times \;R_{\frac{1040}{1400}}+C_{G} \end{array}$$ (17)

Where *S*_*G*_ is classification and purity score; $R_{\frac{1720}{1620}}$ is band intensity ratio of 1,720 cm^−1^ (C=O stretching of carboxylic groups of ketones, aldehydes, and esters) peak to the 1,620 cm^−1^ (aromatic C=C stretching of quinone and conjugated ketone and C=O stretching of and amide groups) peak; $R_{\frac{1040}{1400}}$ is band intensity ratio of 1,040 cm^−1^ (C–O stretching of pothe lysaccharides or polysaccharide-like moieties) peak to the 1,400 cm^−1^ (O–H deformation, C–H deformation of CH_2_ and CH3 groups and phenolic C–O stretching); *C*_*G*_ is classification function constant described by Fuentes et al. (2016). The intensity ratios for $R_{\frac{1720}{1620}}$ and $R_{\frac{1040}{1400}}$ were 0.2214 and 1.5207, respectively. $R_{\frac{1720}{1620}}$ indicates that the aromatic bands are more predominant than carbonyl species, which is a trait of HuA (Zaccone et al., 2006). The *S*_*G*_ for extracted HuA was −22810.68, which was classified as fulvic acid-like HuA (Wang S. et al., 2019). According to Song J. et al. (2024), all HuA species extracted from RS using hydrothermal pretreatment are rich in fulvic acid content (Song C. et al., 2024). Two separate humification indices of the HuA (*HI*_*a*_) and (*HI*_*b*_) was calculated using the following Equations 18 and 19 (Broder et al., 2012). The RS-derived HuA was directly compared with commercial reference HuA (HIMEDIA^®^) (HiMedia Laboratories Private Limited, Mumbai, Maharashtra, India).


$$\begin{array}{l} HI_{a}=\frac{I_{{1520\;cm}^{-1}}}{I_{{1050\;cm}^{-1}}} \end{array}$$ (18)

$$\begin{array}{l} HI_{b}=\frac{I_{{1410\;cm}^{-1}}}{I_{{1050\;cm}^{-1}}} \end{array}$$ (19)

**Figure 7:**
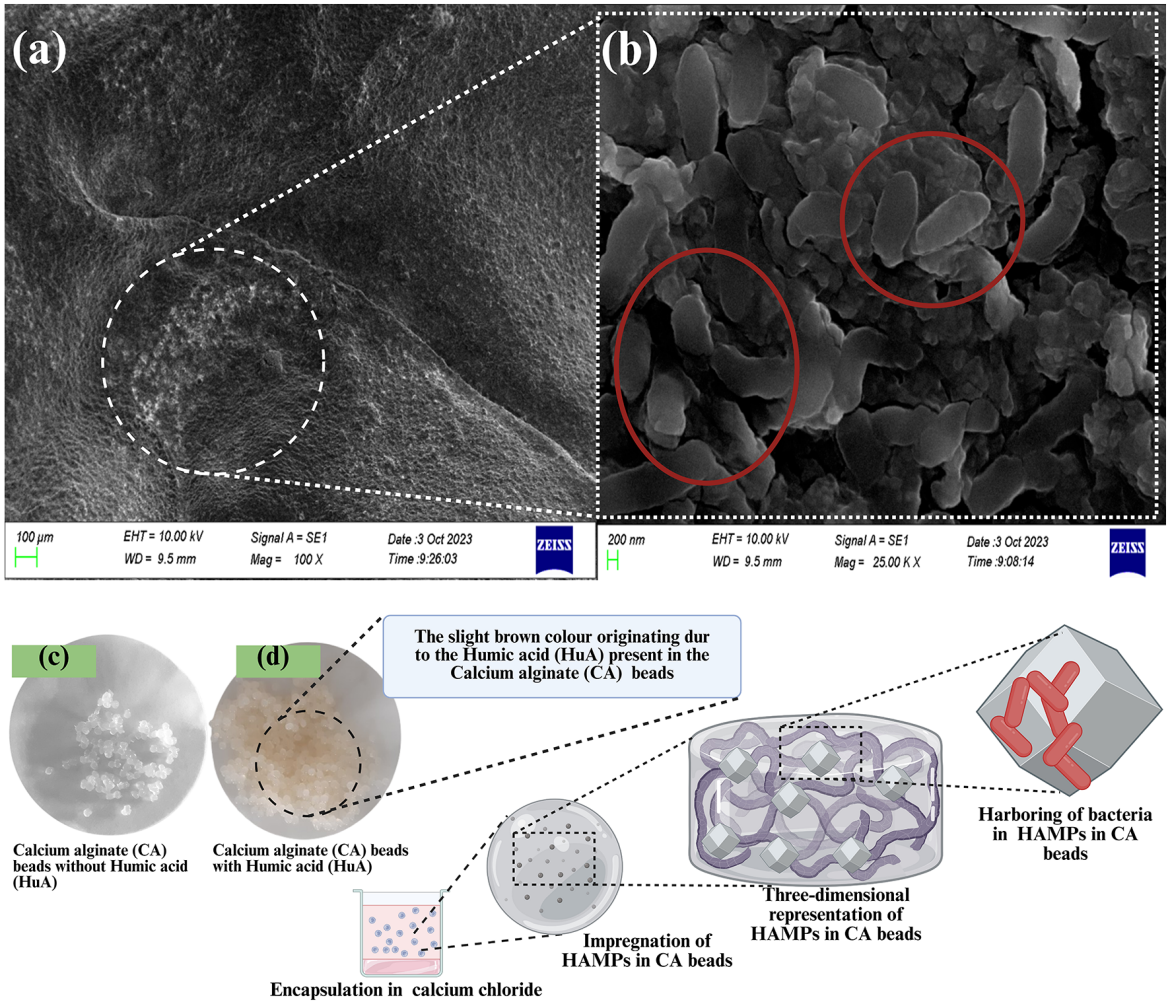
**(a)** SEM micrograph of the surface of bacteria encapsulated calcium alginate beads. **(b)** The colonization of bacterial cells on the surface of the hydroxyapatite nanoparticles dispersed gel bead surface, **(c)** calcium alginate beads without addition of humic acid, **(d)** calcium alginate beads with addition of humic acid and bacterial encapsulation (Zhao et al., 2011).

The *HI*_*a*_ and *HI*_*b*_ for the RS derived HuA was 0.854 and 0.676 and for the reference samples it was 0.761 and 0.618, respectively. The *HI*_*a*_ and *HI*_*b*_ were significantly superior to the commercial reference samples. This observation is consistent with reports by Bharti et al. (2025). The direct comparison of the FTIR spectra of the RS-derived HuA and reference sample is given in Supplementary Figures 2a, b, respectively. Table 2 shows the FTIR analysis of the biocapsule raw material with distinctive peaks characteristic of each material. The successfully embedded bacteria are visible in the SEM image of encapsulated CA beads (Figure 7). The crevices and the hollow regions of HAp nanorod-assembled submicron particles are heavily colonized (Figure 7b).

**Table 2:** FTIR analysis of biocapsule raw materials and synthesized biocapsule components.

Type of biocapsule raw material	Wavenumber (cm^−1^)	Assignment	References
HAp nanorod-assembled submicron particles	461	${\text{PO}}_{4}^{3-}$(υ_2_) bending	Bai et al., 2009; Ciobanu et al., 2010, 2012
	566– 603	${\text{PO}}_{4}^{3-}$ bending (υ_4_)	
	962–1,109	P–O bond stretching of ${\text{PO}}_{4}^{3-}$(υ_1_, υ_3_).	
	3,435	O–H stretching	Bulina et al., 2014; Mansour et al., 2015; Rolo et al., 2014
AC	581	C–H bending of benzene rings	Kong et al., 2013
	940	C–H stretching (alkenes),	Foo and Hameed, 2011
	1,021	symmetric stretching of C–O–C	Thirukumaran et al., 2018
	1,077	C–H in–plane bending	Mohammad and Ahmed, 2017
	1,305	C–O stretching	Ewecharoen et al., 2009
	1,417	C=C ring stretching	Kassahun et al., 2022
	1,600	C=C stretching	Pego et al., 2019
	3,247	O–H stretching (alcohols, phenols, and carboxylic acids)	Rehman et al., 2023
HuA	913	–OH vibration (possible due to kaolinite impurities)	Castellano et al., 2010; Tivet et al., 2013
	1,033	C–N stretching	Dyab et al., 2016
	1,104	C–O stretching	Zhang et al., 2023
	1,388	C–H deformation of CH_2_ and CH_3_, salts of carboxylic acid and/or aliphatic CH	Chien et al., 2007
	1,579	symmetric stretching of amide II; aromatic C = C stretching	Liu et al., 2016; Saxena and Brighu, 2020
	3,414	–OH, –NH stretching	Wang J. et al., 2019
RSOCS	1,035	C–O stretching of AC	Ramadoss et al., 2019
	1,417	C=C ring stretching of AC	Kassahun et al., 2022
	1,419	O–C–H vibration of corn starch	Menchaca-Rivera et al., 2018
	1,631	O–H stretching of water in corn starch	García et al., 2020
	2,925	C–H (stretching vibration) and hydrogen bonds (intra and inter molecular hydrogen bonds) of RS	Basta et al., 2014
	3,430 and 3,433	O–H stretching from moisture, lignin/hemicelluloses, and silica in RS	Basta et al., 2014; Hu and Hsieh, 2014)
	3,694	Inner O–H vibrations of AC	Richmond, 2002
MRH	919	O–H bending hydroxyl of Gly	Shojaei et al., 2024
	1,036	Aliphatic C–O stretching of HuA and C–O stretching of Gly	Baker et al., 2020; Mousa and Taha, 2022
	1,107	C–O–C stretching vibration of Gly	Wu et al., 2021
	1,321	–C–C–H and–O–C–H bending of CMC	Domyati, 2023
	1,416	–COOH– symmetric stretching of CMC	
	1,591	–COOH– asymmetric stretching of CMC	
CA beads	520	P–O–H stretching of HAp nanorod-assembled submicron particle	Sarma et al., 2024
	936	P–O stretching of HAp nanorod-assembled submicron particle	Stella et al., 2022
	1,021	C–O–C stretching of SAs	Zhong et al., 2020
	1,424	Symmetric –C=O stretching of CA	Zhang et al., 2018
	1,604	Symmetric –C=O stretching of CA	
	3,369	Stretching of the O–H in CA	Gao et al., 2023

The purity of the synthesized HAp nanorod-assembled submicron particles using the $CO_{3}^{2-}$ content analyzed through FTIR spectra by a direct comparison of the extinction coefficient (*E*_*i*_) of $CO_{3}^{2-}$ at 1,457 cm^−1^ and $PO_{4}^{3-}$ at 604 cm^−1^ (Murugan et al., 2003).


$$\begin{array}{l} CO_{3}^{2-}\;wt\%=13.5\left(\frac{E_{1457}}{E_{604}}\right)-0.2 \end{array}$$ (20)

Hence,


$$\begin{array}{l} E_{i}\;\left(i=1457\;or\;604\right)=\log\left(\frac{T_{2}}{T_{1}}\right) \end{array}$$ (21)

Where *T*_2_ and *T*_1_ are the transmission intensities of FTIR peak at local baseline.

According to Vetten et al. (2014), the HA carrier produced via wet chemical synthesis (WCS) shows significant aggregation and changes in morphology and can cause contamination when used as microbial carriers. Furthermore, a protocol by Saharan et al. (2010), has used three-time sterilization to ensure that pathogen proliferation does not occur in microbial carriers. Furthermore, Beck (1991) recommends a minimum of three times of sterilization to prevent the prevalence of pathogens in microbial carriers such as HA. Nonetheless, Saharan et al. (2010) recommends keeping safety considerations while performing a number of sterilizations. Therefore, we reduced the number of sterilizations to two times and followed the protocol described by Safari et al. (2020) for our study.

The results indicate that the moisture retention of MRH is increased with HuA incorporation (Zhao et al., 2019). Furthermore, the results indicate that the water solubility of MRH decreases with Gly incorporation (Pan et al., 2008). RS incorporation has increased the RSOCS water solubility alongside AC and CS. The pristine CMC included in MRH recorded a ∅% and ∂ % 6.35% ± 0.39 % and 23.71% ± 1.44%, respectively. Incorporation of Gly into pristine CMC MRH (Gly/CMC) increased ∅% and ∂% up to 28.37% ± 0.88 % and 26.77% ± 0.70%, respectively. Furthermore, a tendency of increase in ∅% and ∂% was shown when HuA was incorporated into the MRH, where ∅% and ∂% reached 33.19% ± 0.36% and 44.91% ± 0.08%, respectively. Furthermore, the ∂% of RSOCS and biocapsule decreased upon incorporation of AC. The ∂% of RSOCS decreased drastically from 12.86% ± 0.65% (RS/CS) to 6.71% ± 1.32% (RS/CS/AC). The biocapsule achieved a ∂% of 56.16% ± 1.65%.

**Figure 8:**
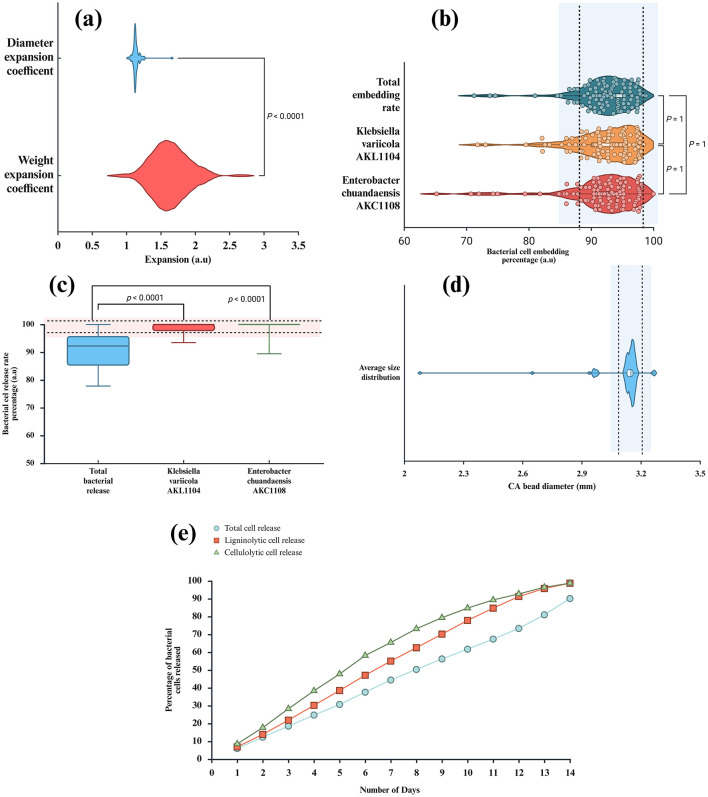
**(a)** Diameter and weight expansion coefficient of calcium alginate beads, **(b)** Bacterial cell embedding of total bacteria, ligninolytic bacteria and cellulolytic bacteria from calcium alginate beads, **(c)** Bacterial cell release rate of total bacteria, ligninolytic bacteria and cellulolytic bacteria from calcium alginate beads, **(d)** Average size distribution of calcium alginate beads, **(e)** percentage of bacterial release from calcium alginate beads.

**Figure 9:**
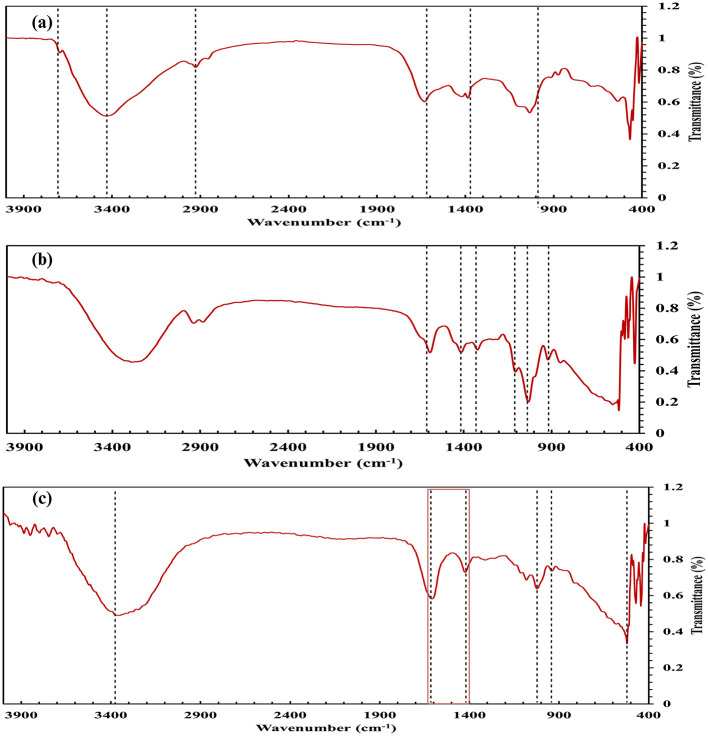
FTIR analysis of the biocapsule raw materials: **(a)** HAp nanorod-assembled submicron particles **(b)**, Activated carbon, **(c)** Humic acid.

**Figure 10:**
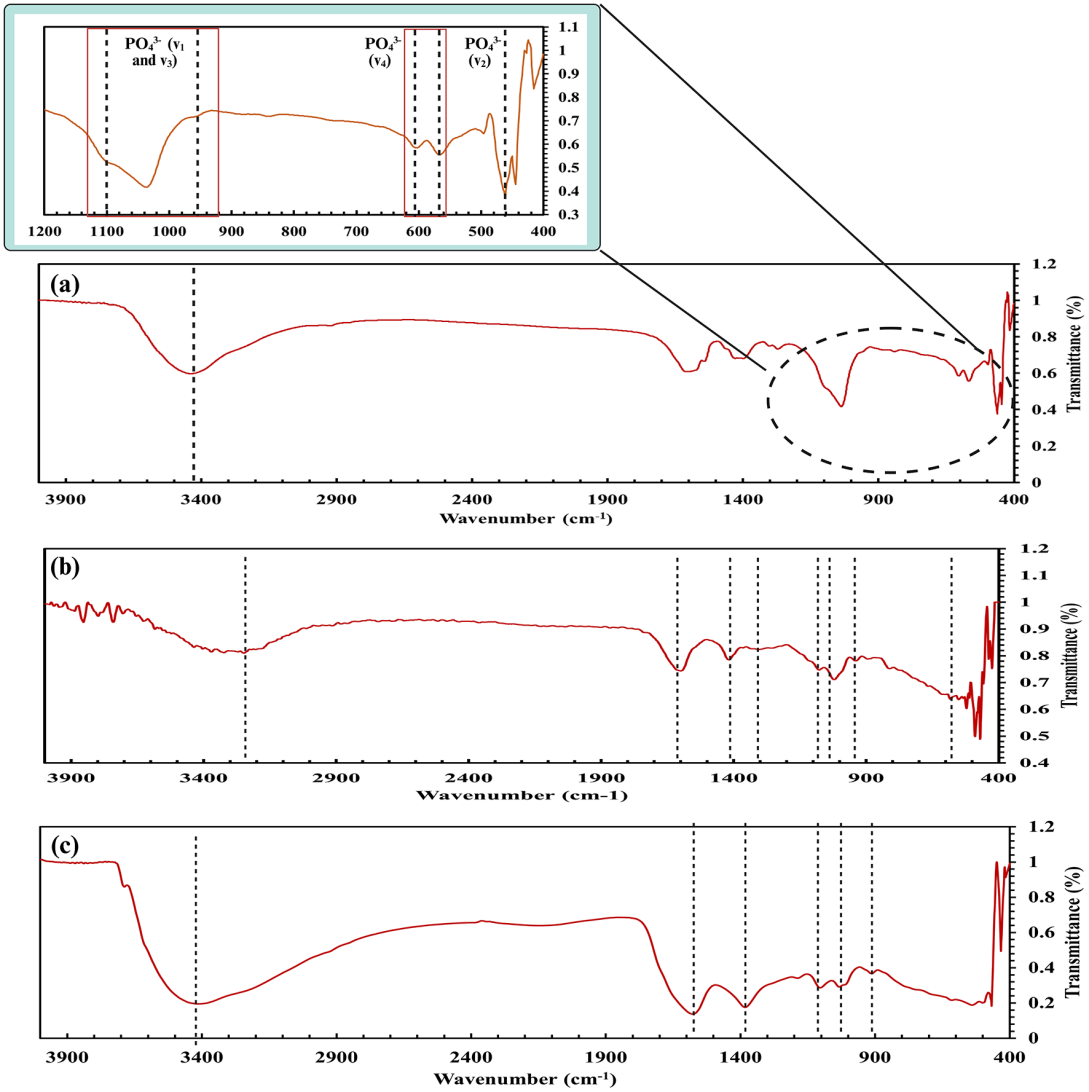
FTIR analysis of the biocapsule components: **(a)** outer rice straw reinforced biocapsule composite shell, **(b)** moisture retention hydrogel, **(c)** Bacteria encapsulated calcium alginate beads.

**Figure 11:**
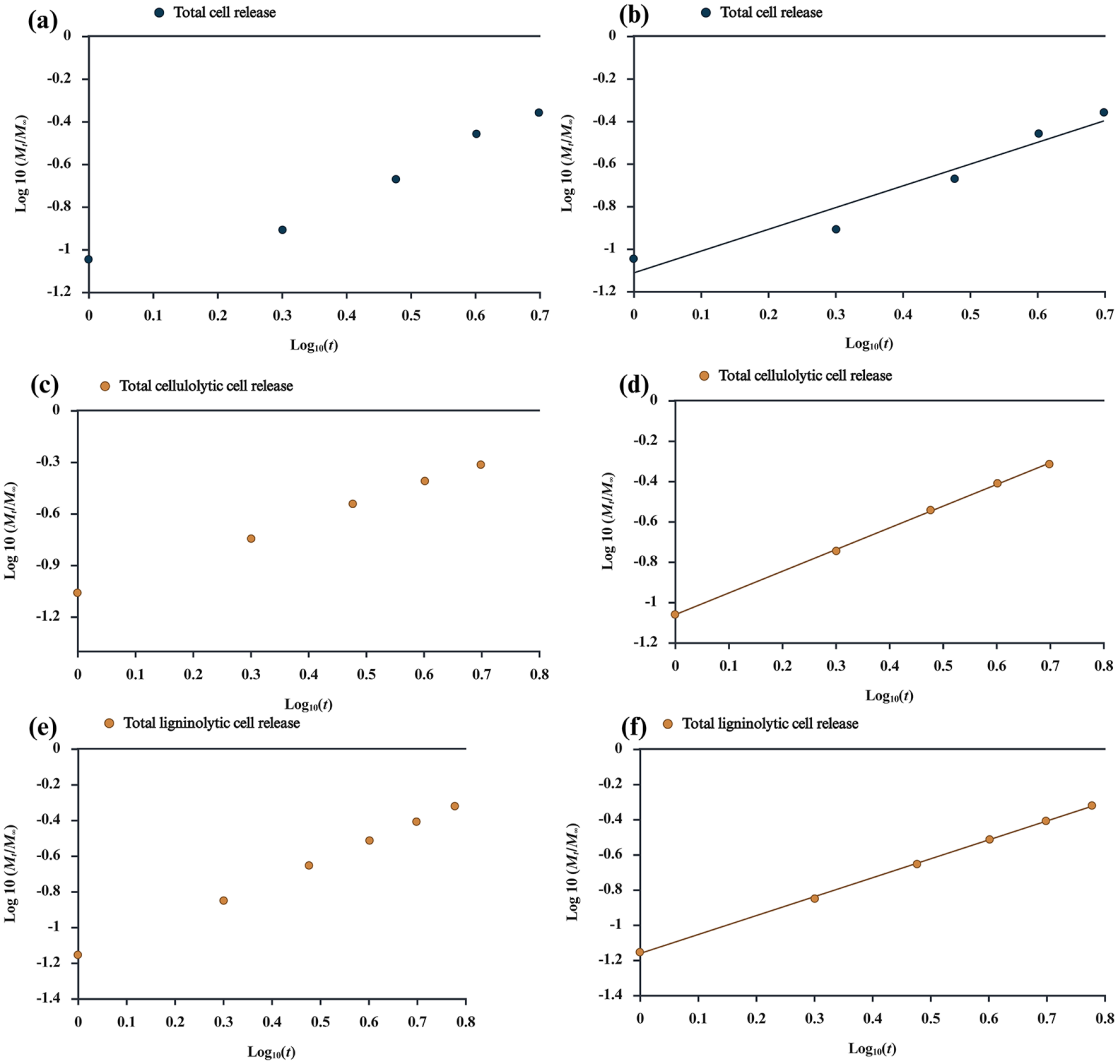
**(a)** Total bacterial release kinetics; **(b)** total bacterial release kinetics with best-fit line; **(c)** cellulolytic bacterial release kinetics; **(d)** cellulolytic bacterial release kinetics with best-fit line; **(e)** ligninolytic bacterial release kinetics; **(f)** ligninolytic bacterial release kinetics with best-fit line.

SEM analysis of RSOCS reveals scattered AC on the surface with fractures on the surface where rice straw reinforcement is inadequate, with a relatively smooth surface due to the plasticizing property of corn starch (Khandanlou et al., 2014) (Figure 6c). The SEM of MRH reveals dense packing and occasional cracks on the surface (Figure 6d). The *WEC* and *DEC* for CA beads, the value was 1.14±0.06 and 1.63 ± 0.27, respectively (Figure 8a). A non-parametric Mann-Whitney test indicated significant interaction between WEC and DEC (*p* < 0.001). The η value (for bacterial encapsulation) (Figure 8b), ζ value (bacterial release) (Figure 8c), σ value (sphericity) of the encapsulated CA beads were 78.29 ± 9.57% and 84.45 ± 6.04% and is 0.222, respectively. There was a significant interaction between total cell release, ligninolytic, and cellulolytic cell release (*p* < 0.001) (Figure 8c). The average size distribution of the CA beads was 3.12 ± 0.12 mm (Figure 8d). However, there was no statistically significant interaction between ligninolytic and cellulolytic bacterial encapsulation in CA (Kruskal–Wallis non-parametric; *p*>0.01). The CA beads released nearly all of the encapsulated total, ligninolytic, and cellulolytic cells to the medium (Figure 8e). Table 3 presents the fitting parameters for the cell release (total cell release, ligninolytic cell release, and cellulolytic cell release). The FTIR analysis of the MRH, RSOCS, and CA beads is shown in Table 2. The FTIR peaks of all the synthesized biocapsule raw materials (HAp nanorod-assembled submicron particles, HuA, and AC) and each biocapsule component are shown in Figures 9, 10, respectively. The cell release kinetics are shown in Figure 11. The release parameters for all three types of bacterial cells are given in Table 3.

**Table 3:** The fitting parameters of the model for bacterial release and the release kinetics parameters.

Type of bacterial release	Equation	R^2^	SD	k	n	D (mm^2^s^−1^)	p value
Total bacterial release	*y* = 1.0223*x* − 1.113	0.9403	0.0778	3.0446	1.0223	0.0900	0.00027
Ligninolytic bacterial release	*y* = 1.0761*x* − 1.163	0.9995	0.0072	3.0535	1.0761	0.0713	6.65874E-05
Cellulolytic bacterial release	*y* = 1.0753*x* − 1.062	0.9996	0.0061	2.8924	1.0753	0.0678	3.1229E-06

The decision to incorporate 13% (w/v) RS fiber and 30% (w/v) cornstarch is based on two approaches: (1) scientific evidence; (2) Thermogravimetric data (TGA) of the composite formulation. Validation of the mechanical strength of RSOCS is vital as it is the primary component that holds the mechanical integrity of the biocapsule (Refer to the images). According to Shoja et al. (2021) and Silva-Guzmán et al. (2018), utilization of 13% (w/v) and 30% (w/v) of RS fiber and corn starch, respectively, enhances the stability of the bio-composites, resulting in increased stiffness and tensile strength. However, during practical use, since the biocapsule will be stored under various conditions, the survivability and stability of the alginate beads and bacterial inoculants under hot storage conditions are important. Furthermore, the next phase of the research is to inoculate the biocapsule into different phases of composting, including the thermophilic phase, which often reaches temperatures of 65–75 °C. It is important to evaluate the thermal stability of the RSOCS. Therefore, we used TGA to evaluate the stability of the RSOCS to gain insights into its ability to protect the inoculants and maintain stability of the gel beads to maintain controlled release of bacteria when inoculated into the thermophilic phase (Huntrakul and Harnkarnsujarit, 2019; Beghetto et al., 2020; Popović et al., 2021; Roy and Rhim, 2020).

Supplementary Figures 3a–d show the TGA data of RSOCS, MRH, and CA components of the biocapsule, respectively, while Supplementary Figure 3b shows the heat transfer within RSOCS during increasing heat. The initial mass loss in RSOCS is the evaporation of water/moisture from the composite. This is further confirmed from the heat flow data of TGA (Supplementary Figure 3b) for the ambient (26.77 °C) to 100 °C temperature range. The heat flow for this range of this temperature range is negative and therefore endothermic. The rapid initial mass loss in MRH is due to surface moisture loss, and the mass loss near 100 °C is due to water loss embedded in the CMC matrix (Roy and Rhim, 2020). The mass loss of around 200 °C in CA beads is due to the breakdown of sodium alginate.

### 3.2 Biodegradability, biocompatibility, and interlayer interactions of biocapsule components

**Figure 12:**
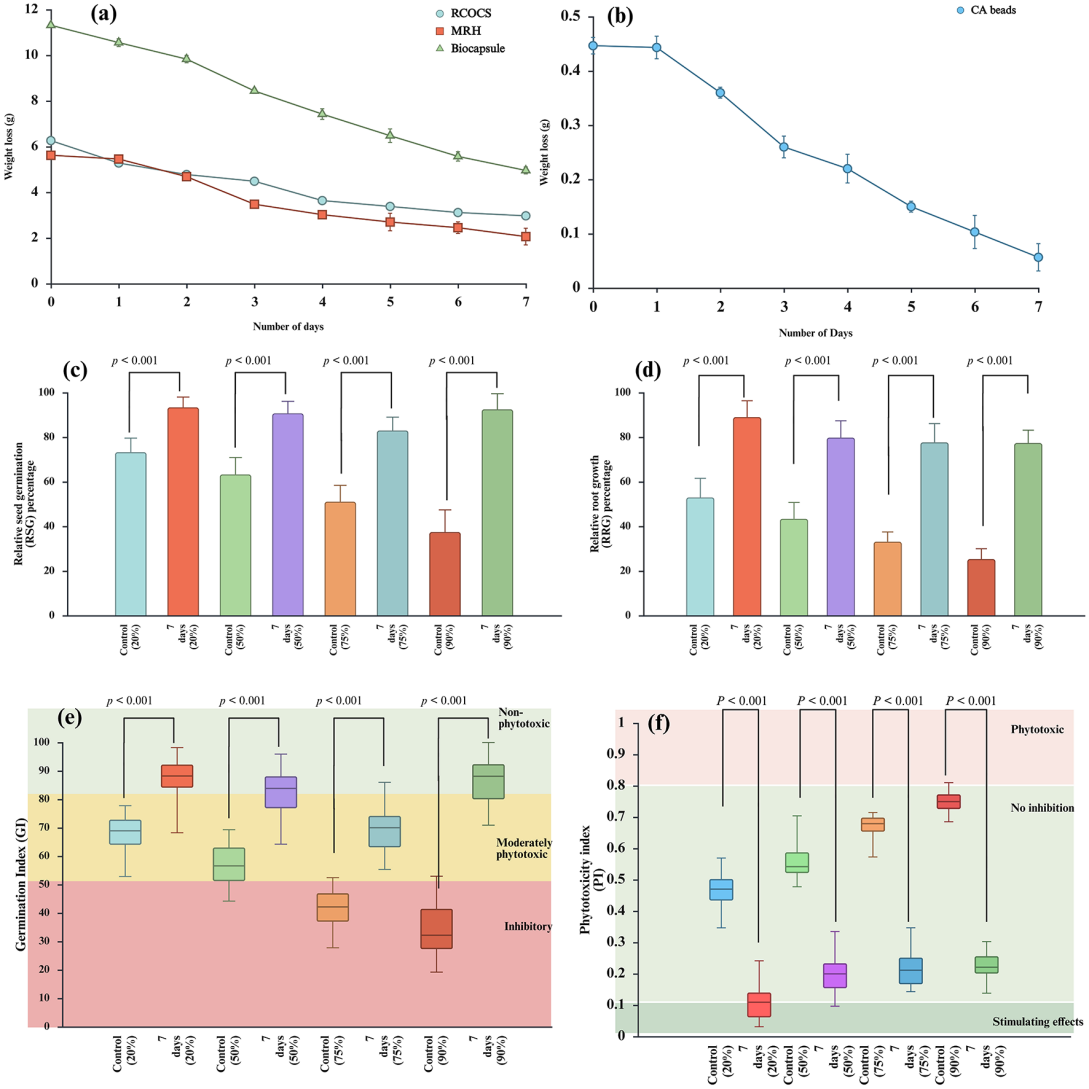
**(a)** Relative seed germination percentage, **(b)** Relative root growth, **(c)** Germination index, **(d)** Phytotoxicity index. All the parameters are measured against a control (0 days) and test samples (7 d of duration), **(e)** Biodegradability of moisture retention hydrogel, outer rice straw reinforced biocapsule composite shell and entire biocapsule, **(f)** Biodegradability of bacteria encapsulated calcium alginate beads.

During biodegradation studies, the RSOCS, MRH, and CA beads recorded degradation efficiency of 52.54% ± 2.69%, 63.33% ± 6.30%, and 87.40% ± 5.41%, respectively. The entire capsule showed degradation efficiency of 56.16% ± 1.65% within 7 d (Figures 12a, b). The biodegradation of RSOCS, MRH, and CA beads was statistically significant with time (*p* < 0.001) (a non-parametric Mann-Whitney test). At the end of 7d, the ψ for the biocapsule 93.41 ± 4.61%, 90.73% ±5.36%, 83.03% ± 5.98%, 92.48% ± 7.04% respectively for all the concentrations (20%, 50%, 75%, 90%). At 7d, the λ reached for all the concentrations were 0.10 ± 0.07, 0.20 ± 0.07, 0.22 ± 0.08, 0.22 ± 0.05, respectively for all the concentrations (20%, 50%, 75%, and 90%). At the end of 7 days, *GI* for the biocapsule degradation were 83.23% ± 8.38%, 72.41% ± 7.64%, 64.38% ± 7.43% and 71.61% ± 7.56%, respectively for 20%, 50%, 75%, and 90% digestate concentrations (Figures 12c–f).

MRH moisture retention improves from 6.35% ± 0.39% (*CMC*) to 28.37% ± 0.88% (*CMC*+*Gly*) to 33.19% ± 0.36% (*CMC*+*Gly*+*HuA*), and provides a cushioning effect against desiccation as the accumulated water creates a thermal reservoir where the high specific heat capacity of water prevents evaporation from the MRH and preserves the moisture for the inoculants. The interlayer FTIR analysis reveals that new CMC-alginate hydrogen bonds at 3,311 *cm* and HuA-mediated *Ca* chelation to CMC at 1,590 *cm* for formed. The formation of *Ca*− mediated crosslinking suggests that the entrapment of moisture via hydrogen bonding is feasible in the *MRH*−*CA* interface (Bher et al., 2022; Lima et al., 2021). The FTIR analysis of the interlayer compatibility is shown in Table 4.

**Table 4:** Interlayer compatibility within the biocapsule layers and evidence of new bond formation.

Interlayer interface	Wavenumber (cm^−1^)	Assignment	References
CA-MRH	3,311	New peak for hydrogen bond formation in –OH– groups of CMC and alginate chains	Henao et al., 2018
	2,921	Formation of methyl C–H bond between HuA in CA beads and glycerol and CMC in MRH	Lu et al., 2019
	1,590	HuA crosslinking to CMC in MRH via multipoint chelation between –COOH– of CMC and *Ca*^2+^ ion	Lu et al., 2019
	1,320	In-plane bending of C–H in CH=CH and CH_2_ groups of CMC in MRH, which causes the crystallization of metal ions such as *Ca*^2+^	Emam et al., 2019
RSOCS-MRH	3,436	O–H vibrations originating from microcrystalline cellulose in RS and CMC of MRH	Çelikçi et al., 2022; Lisboa et al., 2021; Zheng et al., 2023
	2,934 and 2,884	Reduction of the twin peaks of CH_2_ and methine (CH) stretching vibrations of CMC in MRH due to polymerization reactions between AC and CMC in MRH	Wang et al., 2022
	1,067 and 1,021	The C–O stretching vibrations of corn starch in RSOCS and CMC in MRH due to ester bridge formation between CMC of MRH and the starch aldehydes of RSOCS	Lee et al., 2018
RSOCS-CA	3,694	Adsorbed water into *Ca*^2+^ in CA	Ward, 1968
	1,638	O–H bending of adsorbed water into the RS of RSOCS	Chen et al., 2022
	3,528	O–H stretching vibration due to crosslinking of free hydroxy moieties in starch in RSOCS and *Ca*^2+^ in CA	Wei et al., 2025
	3,451	O–H stretching vibration due to hydrogen bonding of COOH groups in alginate in CA and O–H groups of corn starch in RSOCS	Su et al., 2022; Wei et al., 2025
	3,376	Bond shift from 3,410 to a lower wavenumber indicates increased hydrogen bonding within CA beads	Ghanbari et al., 2022
	1,639	O–H stretching vibration of adsorbed water into HAp nanorod-assembled submicron particles overlapping with asymmetric stretching vibration of alginate in CA beads	Akshata et al., 2023

The SEM micrographs of the CA-MRH junction showed a rough, bead-like surface on the MRH, where the smooth plasticizing characteristic of Gly diminished. The large globules on the MRH surface might be microaggregates of CA particles, and the alkoxy radicals on the CMC of MRH and CA microaggregates can cause cross-linking and bridge formation, as shown in Supplementary Figure 4 (Chen and Zhao, 2000; Pourjavadi et al., 2006). Further magnification of the other regions of MRH shows that the ridges of the MRH have been colonized by the bacteria (Supplementary Figure 4a). This can be attributed to the CA-CMC biocompatibility, where the entrapped bacteria migrate from the alginate matrix to the CMC via the cross-linking bridges. SEM micrograph of the RSOCS-MRH interface shows the direct attachment of calcium alginate particles to the MRH surface alongside the rice straw particles in RSOCS. The MRH surface is still smooth, as it signifies that a substantial amount of moisture has been retained to keep the smoothness of MRH (Supplementary Figure 4b). SEM micrograph of the RSOCS-CA interface shows the crystallized HAp nanorod-assembled submicron particles attached to the RSOCS surface, while the RSOCS surface has become wrinkled due to loss of moisture, and also the globular nature observed in the freshly prepared RSOCS surface has disappeared due to the degradation of corn starch on the surface (Supplementary Figure 4c).

### 3.3 Composting analysis

#### 3.3.1 Compost fertility and nutrient availability

**Figure 13:**
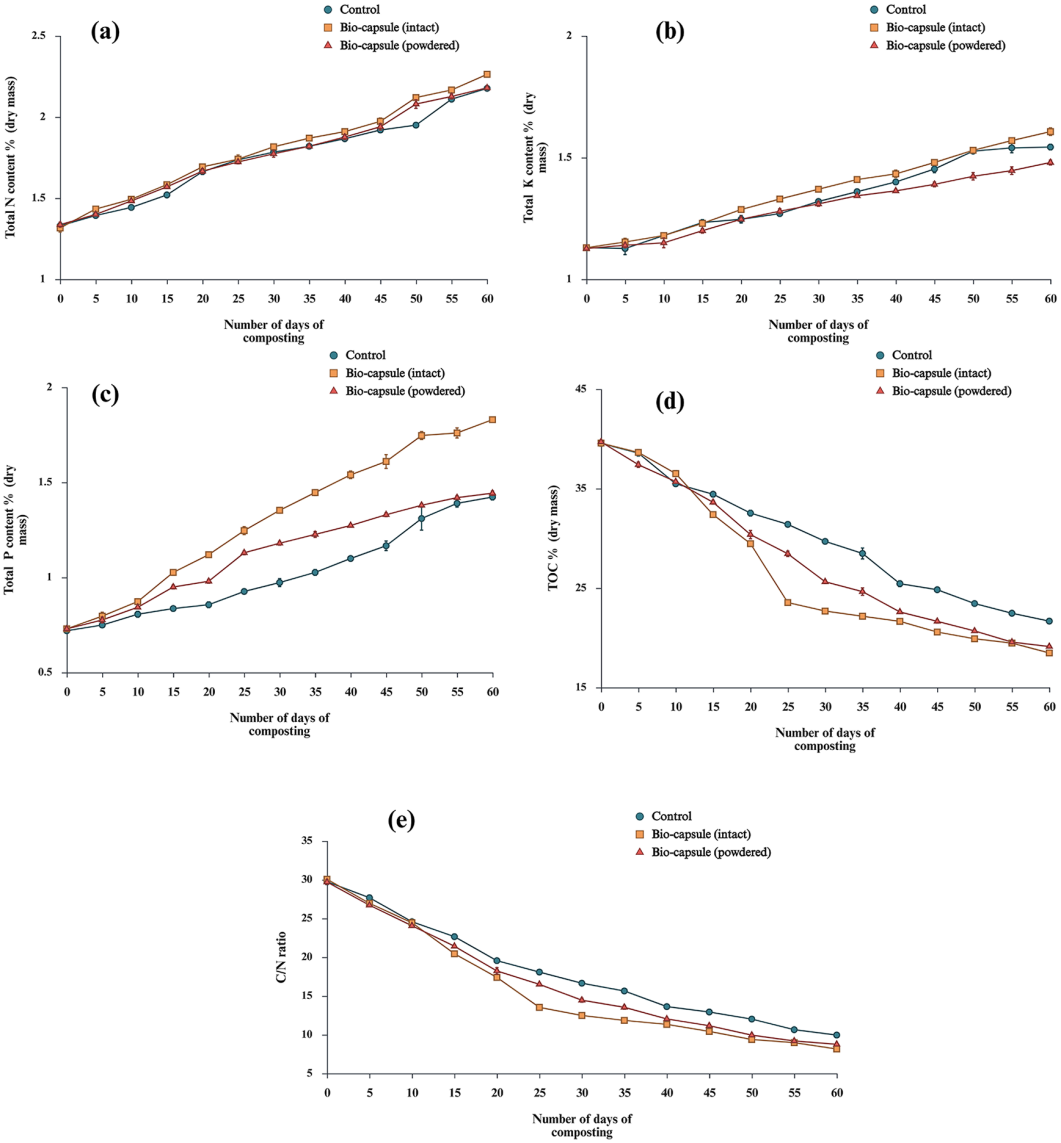
**(a)** Total N content % fluctuations by dry mass during composting, **(b)** Total K content % fluctuations by dry mass during composting, **(c)** Total P content % fluctuations by dry mass during composting, **(d)** Total organic carbon content % fluctuations by dry mass during composting, **(e)** C/N ratio fluctuations during composting.

The total increase in *TN*_*dm*_% for $T_{biocapsule}^{S},\;T_{biocapsule}^{PS}$ and *T*_*control*_ were 71.89%, 63.09% and 63.65%, respectively. After 25d, the mean rate of *TN*_*dm*_% increase was significant in $T_{biocapsule}^{S}$ (0.66*% day*^−1^) compared to $T_{biocapsule}^{PS}$ (0.59*% day*^−1^ and *T*_*control*_ (0.57*% day*^−1^) (*Tukey*′*s p* ≤ 0.01;α = 0.05). This might be an indication of the effectiveness of the biocapsule technology in lignocellulosic composting. The *TN*_*dm*_%, increase in all treatments was statistically significant (*p* < 0.01) (Figure 13a). The *TK*_*dm*_% increased in and the overall *TK*_*dm*_% is statistically significant in all three treatments. The mean percentage of increase of *TK*_*dm*_% at the end of composting are 60.66%, 35.33% and 54.33% for $T_{biocapsule}^{S},\;T_{biocapsule}^{PS}$ and *T*_*control*_, respectively. The mean rate of *TK*_*dm*_% increase for each treatments showed a hierarchy of $T_{biocapsule}^{S}>T_{control}>T_{biocapsule}^{PS}$ (*Tukey*′*s p* < 0.01) (Figure 13b). The overall mean *TP*_*dm*_% increase after 60 d composting for $T_{biocapsule}^{S},\;T_{biocapsule}^{PS}$ and *T*_*control*_ are 1.83%, 1.44%, and 1.42%, respectively, compared to the initial 0.72%. The mean *TP*_*dm*_% increase percentage after composting are 83.0%, 44.33%, and 42.33%, respectively, for $T_{biocapsule}^{S},\;T_{biocapsule}^{PS}$ and *T*_*control*_, where $T_{biocapsule}^{S}$ had a significantly higher *TP*_*dm*_% after 60d. The overall *TP*_*dm*_% is statistically significant (*p* < 0.01) among all three treatments. The end *TP*_*dm*_% in $T_{biocapsule}^{S}$ is statistically significant compared to $T_{biocapsule}^{PS}$ and *T*_*control*_ (*Tukey*′*s p* < 0.01). Furthermore, ANOVA reveals that the rate of *TP*_*dm*_% increase was statistically significant in $T_{biocapsule}^{S}$ compared to $T_{biocapsule}^{PS}$ and *T*_*control*_ (*p* < 0.01; *Tukey*′*s p* < 0.01) (Figure 13c). The *TOC*_*dm*_%, showed gradual decline in all the three composting treatments. The *TOC*_*dm*_%, reduction in $T_{biocapsule}^{S},\;T_{biocapsule}^{PS}$ and *T*_*control*_ were 53.30%, 51.83%, and 45.19%, respectively. However, it can be clearly observed that from 10 d to 25 d, the rate of *TOC*_*dm*_%, reduction in $T_{biocapsule}^{S},\;T_{biocapsule}^{PS}$ is significantly higher than in *T*_*control*_. The mean rate of *TOC*_*dm*_% reduction in this period is 0.023*% day*^−1^, 0.013*% day*^−1^ and 0.007*% day*^−1^ for $T_{biocapsule}^{S},\;T_{biocapsule}^{PS}$ and *T*_*control*_, respectively. The decrease of *TOC*_*dm*_% was statistically significant in all the treatments (*p* < 0.01). However, the overall *TOC*_*dm*_% decrease between $T_{biocapsule}^{S},\;T_{biocapsule}^{PS}$ was not significant (*p*>0.01) (Figure 13d). *CN* ratio decreased in all three treatments during the composting period where the initial and final *CN* ratio for $T_{biocapsule}^{S},\;T_{biocapsule}^{PS}$ and *T*_*control*_ are 30.06 and 8.16, 29.70, and 8.77, 29.74 and 9.96, respectively. The *CN* ratio decline was statistically significant in all the three treatments (*p* < 0.01). At the end of 60d, the mean percentage of *CN* decline for $T_{biocapsule}^{S},\;T_{biocapsule}^{PS}$ and *T*_*control*_ were 72.83%, 70.47%, and 66.51%, respectively. The $T_{biocapsule}^{S}$ had the lowest *CN* which is significantly lower than $T_{biocapsule}^{PS}$ and *T*_*control*_ (*Tukey*′*s p* < 0.01). The rate of *CN* was statistically significant in all three treatments in a manner where $T_{biocapsule}^{S}>\;T_{biocapsule}^{PS}$ > *T*_*control*_ (*all pairwise Tukey*′*s p* < 0.01) (Figure 13e).

#### 3.3.2 Composting conditions and dynamics

**Figure 14:**
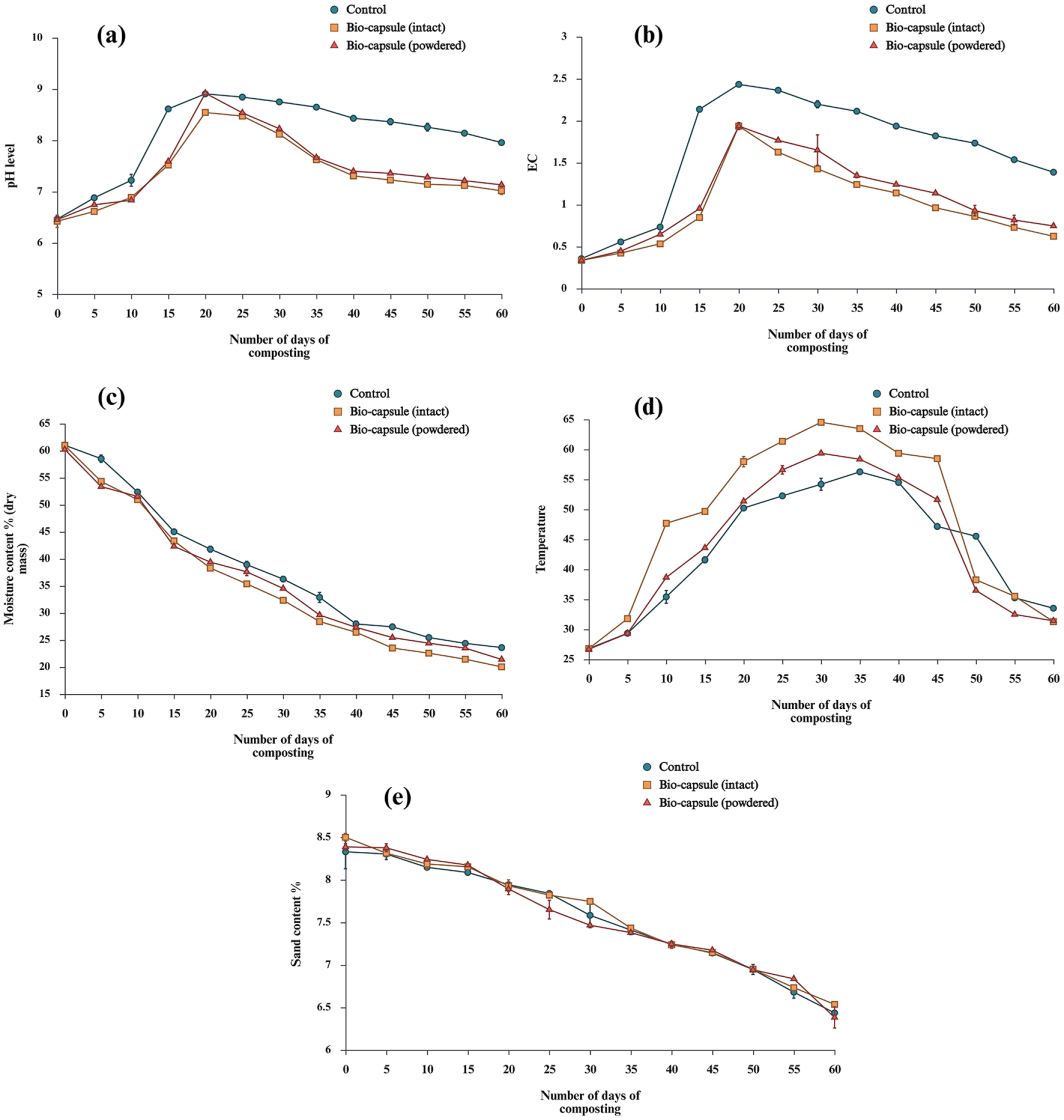
**(a)** pH level fluctuations during composting, **(b)** Electric conductivity fluctuations during composting, **(c)** Moisture content % fluctuations by dry mass during composting, **(d)** Temperature profile fluctuations during composting, **(e)** Sand content % fluctuations during composting.

A rapid rise in the pH is seen from 15 to 20 d in all three treatments. The initial average pH of $T_{biocapsule}^{S},\;T_{biocapsule}^{PS}$ and *T*_*control*_ are 6.42, 6.45, and 6.46, respectively (slightly acidic). The peak pH was reached to alkaline levels due to ammonification (see discussion). The maximum average pH reached during the ammonification phase are 8.54, 8.92, and 8.90 $T_{biocapsule}^{S},\;T_{biocapsule}^{PS}$ and *T*_*control*_, respectively. The pH at the end of the composting trial reached a pH of 7.01, 7.13, and 7.95. The composting pH reached neutrality in two treatments ($T_{biocapsule}^{S}$ and $T_{biocapsule}^{PS}$) except for *T*_*control*_. The one-way ANOVA shows significant differences among treatments (*p* < 0.05) (Figure 14a). The EC showed a rapid rise from 10d to 20d in $T_{biocapsule}^{S},\;T_{biocapsule}^{PS}$ and *T*_*control*_. The average initial EC were 0.33 *DS ml*^−1^, 0.33 *DS ml*^−1^, and 0.35 *DS ml*^−1^ for $T_{biocapsule}^{S},\;T_{biocapsule}^{PS}$ and *T*_*control*_, respectively. The maximum EC was reached at ~20d, which is 1.93 *DS ml*^−1^, 1.93 *DS ml*^−1^and 2.43 *DS ml*^−1^ for $T_{biocapsule}^{S},\;T_{biocapsule}^{PS}$ and *T*_*control*_ respectively. The average final EC was recorded as 0.62 *DS ml*^−1^, 0.74 *DS ml*^−1^ and 1.46 *DS ml*^−1^ for $T_{biocapsule}^{S},\;T_{biocapsule}^{PS}$ and *T*_*control*_, respectively. The one-way ANOVA shows significant differences among treatments (*p* < 0.05) (Figure 14b).

In 60 days, *MC*_*dm*_% for $T_{biocapsule}^{S},\;T_{biocapsule}^{PS}$ and *T*_*control*_ was 21.3% ± 0.1%, 20% ± 0.4%, and 23.5% ± 0.4%, respectively. The *MC*_*dm*_% has shown a gradual decline in all three treatments. The results were consistent with reports of (Zhu 2007). The *MC*_*dm*_% reduction among treatments was statistically significant (*p* < 0.01). $T_{biocapsule}^{S}$ showed highest *MC*_*dm*_% reduction rate throughout composting. Tukey's HSD indicates the degree of *MC*_*dm*_% reduction as $T_{control}<T_{biocapsule}^{PS}<\;T_{biocapsule}^{S}$ (all pairwise *p* < 0.01;α = 0.05). A higher degree of moisture loss in $T_{biocapsule}^{S}$ (~ 67.15%) and $T_{biocapsule}^{PS}$ (~ 64.46%) was observed compared to *T*_*control*_ (~ 61.32%) (Figure 14c).

The onset of the high-temperature phase varied among all the treatments. The $T_{biocapsule}^{S}$ reached the high-temperature phase on 20d, reaching a maximum temperature of 64.53 ± 0.21 °C on 30d while sustaining temperatures above 55 °C for 25 days. The importance of maintaining a pile temperature above 55 °C is explained later in the discussion. $T_{biocapsule}^{PS}$ reached high-temperature phase on 25 d and peaked at 59.37 ± 0.29 °C on 30d and sustained high temperature above 55 °C for 15 d while *T*_*control*_ reached peak temperature of 56.27 ± 0.15 °C lasting for a single day. The temperature fluctuations were statistically significant in all three treatments (*p* < 0.01). The whole-temperature profile analysis at the replicate level $T_{biocapsule}^{S}$ yielded the highest thermophilic stage exposure in AUC (Area Under Curve), followed by $T_{biocapsule}^{PS}$ and *T*_*control*_. The mean temperature, mean peak temperature, and AUC for all three treatments indicated a significant effect on the type of inoculation ($T_{biocapsule}^{S},\;T_{biocapsule}^{PS}$ and *T*_*control*_ (no inoculant externally introduced) influenced thermogenesis and heat retention during composting (η^2^≈ 0.998 ;*p* < 0.01) (Figure 14d). The (SLS) 1636: 2019 mandates the *S%* mandates to be below 20%, studies where the *S%* is actively monitored throughout the composting period are hardly found. Nonetheless, the initial mean *S%* of the for $T_{biocapsule}^{S},\;T_{biocapsule}^{PS}$ and *T*_*control*_ are 8.50%, 8.38%, and 8.33%, respectively. The overall *S%* of the treatment was not significantly reduced during the composting period. All the treatments showed a mean observable decrease of $S\%\;(T_{biocapsule}^{S};6.53\%,\;T_{biocapsule}^{PS};6.38\%$ and *T*_*control*_; 6.43%). Nonetheless, this trend was not statistically significant among the treatments (*p*>0.01) (Figure 14e). Furthermore, Table 5 shows the SLS 1635:2019 standards against the characteristics of the final products of each treatment.

**Table 5:** SLS 1635:2019 requirements comparison with the final products of all composting treatments.

Compost characteristics	Final product (mean ±SD)			SLS requirement
${\text{T}}_{\text{b}iocapsul\text{e}}^{\text{S}}$	${\text{T}}_{\text{b}iocapsul\text{e}}^{\text{P}\text{S}}$	T_control_	
pH	7.01 ± 0.07	7.13 ± 0.02	7.95 ± 0.02	6.5–8.5
EC	0.62 ± 0.00	0.74 ± 0.01	1.46 ± 0.02	< 4
*MC*_*dm*_%	20.03 ± 0.46	21.40 ± 0.14	23.6 ± 0.41	< 25%
*TN*_*dm*_%	2.26 ± 0.01	2.18 ± 0.01	2.17 ± 0.00	>1%
*TP*_*dm*_%	1.83 ± 0.01	1.44 ± 0.01	1.42 ± 0.01	>0.5%
*TK*_*dm*_%	1.60 ± 0.01	1.48 ± 0.01	1.54 ± 0.01	>1%
*TOC*_*dm*_%	18.48 ± 0.16	19.12 ± 0.02	21.68 ± 0.16	>20%
*CN*	8.16 ± 0.11	8.77 ± 0.03	9.96 ± 0.05	10–25
*S%*	6.53 ± 0.02	6.38 ± 0.12	6.43 ± 0.01	< 20

## 4 Discussion

### 4.1 Characterization of the biocapsule raw materials and individual biocapsule components

During SEM analysis, the surface morphology of HAp nanorod-assembled submicron particle shows plate-like regions with distinct boundaries between packed nanorods (Figure 4b). In some regions, uniform clusters of chrysanthemum-like micro-flowers are visible (Figure 4b). The HAp nanorod-assembled submicron particle possesses an average size of 525 nm for HAp nanorod-assembled submicron particle (Figure 4c). According to Zhang et al. (2009), HA particle formation reaction with citrate ions (as described in the protocol) is as follows.


$$\begin{array}{c} 10{\left[Ca\;Cit\right]}^{-}+{6PO}_{4}^{3-}+{2OH}^{-}+Ca_{10}{\left(PO_{4}^{3-}\right)}_{6}{\left(OH\right)}_{2}↓ \end{array}$$

$$\begin{array}{c} +10Cit^{3-} \end{array}$$ (22)

*Cit*^3−^ions are selectively adsorbed into various hexagonal facets of the growing HA crystal and cause relative surface energy fluctuations (Zhang et al., 2009). Therefore, the growth rate shifts toward certain orientations and leads to HA nanorod production (Refer to the SEM image) (Hu et al., 2011). According to Rodríguez-Clemente et al. (1998), the self-aggregation of HA into submicron particles has three steps: (1) nucleation reaction and crystal formation in nano range; (2) elemental nanocrystal aggregation by physical attraction; (3) enhanced crystal growth at a constant residual supersaturated condition and acts as the cementing point inside the HA aggregate to form a stabilized agglomerate. The agglomerates grow up to the submicron level, as observed in the particle size of the synthesized HA particles. Furthermore, particle size increase takes place where the sub-micron level agglomerates form secondary agglomerates in micrometric range. According to Gomez-Morales et al. (2001), HA aggregation is governed by surface free energy minimization where electrical surface charge causes the submicron level HA particle aggregation.

During FTIR analysis, 4.95 ± 0.05 *wt%* was obtained as the $CO_{3}^{2-}\;wt\%$ for the synthesized HAp nanorod-assembled submicron particles, which is highly close to the $CO_{3}^{2-}\;wt\%$ range of 3–8 *wt%* for pure biological HAp particles. According to Nga et al. (2018), this ratio is highly similar to biological HAp nanorod-assembled submicron particles and resembles biological HAp nanorod-assembled submicron particles in terms of $CO_{3}^{2-}\;wt\%$. Furthermore, the SEM-EDAX analysis reveals a *Ca*/*P* ratio of 2.08 for HAp nanorod-assembled submicron particles. This exceeds the stoichiometric ratio of 1.67 for HAp nanorod-assembled submicron particles. This can be attributed to Ca-rich material precipitation from the *Cit*^3−^ solution and similar reports indicate that formation of an amorphous phase may contribute to the excess Ca content, which can contribute to *Ca*/*P* ratio between 1.2 and 2.5 (LeGeros, 2001) (Figure 5a).

The smaller particles in HuA might assist in high metal absorption for bacterial proliferation during the release of inoculants in the feedstock. The HuA yield from RS was 16.6% ± 0.003%. During the HuA SEM analysis, compacted microaggregates are visible (Figure 5). This aggregation might assist in essential metal ion transport for bacterial inoculants during biodegradation of feedstock (Chen et al., 2007; Pandey et al., 2000; Xu D. et al., 2006). Thin thread-like structures are visible, which might grow into larger sheets and rings (Figure 6a). Utilization of a high concentration of KOH (10%) allows the formation of multilayered aggregations in HuA with a porous surface (evident in SEM images) (Yuan et al., 2017). High concentration of KOH allows K chelation with –COO– and –OH– groups in HuA, providing atomic-level dispersion, which ultimately leads to particle formation with increased porosity (Chassapis et al., 2009; Garcia et al., 1993; Paciolla et al., 1999). During AC formation, the dissolution of lignin and other mineral compounds during activation, which ultimately causes micro and mesopore formation in the coconut shells (Figure 6c) (Oh and Park, 2002).

According to Kaschl and Chen (2005), Mirza et al. (2011), Tahir et al. (2010), De Melo et al. (2015), and Hajdrik et al. (2022), HuA forms micelle-like structures in neutral aqueous conditions, which can improve the water retention in hydrogels. Gly incorporation in MRH, increased the moisture retention, as Gly enhances water and Oxygen permeability (Jouki et al., 2013). Despite the inherent hydrophilic characteristics of CMC due to rigid and long carbon chains and abundance of free –OH moieties, which ultimately form firm intra- and intermolecular H bonding between carbon chains, Gly incorporation increased the water solubility of MRH (Pandey et al., 2014). According to Li et al. (2018), Wang et al. (2018), and Nguyen et al. (2020), the hydrophobicity of rice straw fibers reduces the water solubility of RSOCS. Furthermore, the homogenous embedding of hydrophilic AC in the CS matrix in RSOCS also contributes to reduced water solubility (Kaur and Thirugnanam, 2016).

The dense packing in MRH in SEM analysis can be attributed to the non-homogeneity and large molecular size of CMC fibrils (Figure 6d). The observations are in supported by previous study by Martins et al. (2020). The addition of glycerol has reduced the crack formation and reduced membrane roughness (Jouki et al., 2013; Khan et al., 2020; Li et al., 2021). FTIR analysis of CA beads with encapsulated bacteria does not reveal the characteristic peaks in HAp nanorod-assembled submicron particle which is assumed to be diminished due to the low concentration. The high SA (3%) concentrations allow to achieve higher *WEC* and *DEC* which causes a sustained and controlled release of entrapped bacteria. This can be attributed to the high-water absorbent ability of the CA beads. The high η value (for bacterial encapsulation) for encapsulated CA can be attributed to the high concentration of SA. Likewise, the high ζ value can be attributed to the high SA % which causes elevated water absorption (swelling) and disrupts the polymer configuration of CA and ultimately releases the entrapped cells. According to Tran et al. (2024), low σ values (sphericities < 0.05) enhances the mechanical stability of the CA beads. Therefore, CA beads can be kept stable for a long time inside the biocapsule. Since both bacterial isolates are Gram negative, humic acid does not interact with the peptidoglycan layer in the cell wall cause cell damage (Song J. et al., 2024; Zhang et al., 2024).

RSOCS is capable of sacrificing the evaporation of water molecules to prevent the heat transfer into the interior of the biocapsule where the bacterial inoculants are stored. It can be attributed to the moisture leaving RSOCS by absorbing the increasing heat flow to prevent the bacterial death due to sudden heat exposure of CA beads. Furthermore, the RSOCS is able to prevent the CA bead degradation in thermophilic phase and in high-temperature storage environments up to 250 °C (Supplementary Figure 3a) (Beghetto et al., 2020; Huntrakul and Harnkarnsujarit, 2019; Roy and Rhim, 2020). Therefore, the RSOCS composition of 13% (w/v) and 30% (w/v) was utilized due to the thermal protection offered by this composition and confirmed scientific evidence. Nonetheless, the thermal protection offered by RSOCS allow the sodium alginate matrix to be intact without experiencing temperature increases which might affect the viability of the bacteria (Beghetto et al., 2020; Huntrakul and Harnkarnsujarit, 2019). The thermal buffering induced by RSOCS via an endothermic moisture-loss window from ~26.8 °C−100 °C absorbs heat during thermogenesis and limits the heat transfer to CA beads and keep the alginate below thermal transition.

### 4.2 Bacterial cells release kinetics and bacterial interactions with the microbial carrier

During this study, the bacteria encapsulated beads can be visualized as a network of alginate and HAp nanorod-assembled submicron particles where HAp nanorod-assembled submicron particles with embedded bacteria on its surface as present in voids (Figure 7). It is speculated that the when CA beads are introduced into the release medium, water molecules enter the beads and initiates polymer swelling and ultimately lower the glass transition temperature and cause network chain loosening. According to Roy et al. (2008), the bacterial cells in CA beads gets released into the penetrant water molecules and gets diffused into the medium with the following possibilities: (1): When the rate of bacterial release is slower than the rate of polymer chains, the bacterial release mechanism is Fickian; (2): When the rate of polymer relaxation is slower than bacterial diffusion, the mechanism is Non-Fickian; (3) Intermediate state where the bacterial diffusion is almost as same as the polymer chain relaxation or anomalous in nature. It is clearly visible that *n* values for all types of cell release are above 1, which means that the cell release mechanism is Case II mechanism. According to Roy et al. (2008), the *n* values close to 1 or more than 1 is considered as the Case II mechanism which is the most desired condition for the controlled release.

To analyze the microbial interactions between the HAp nanorod-assembled submicron particles and bacterial inoculants, the HAp nanorod-assembled submicron particles were incubated for 30 d (half of the composting time period of the biocapsule) at 37 °C without shaking in LB agar medium. The HAp nanorod-assembled submicron particles were separated from the medium via centrifugation at 15,000 RPM for 15 min and were subjected to freeze drying overnight. The samples were subjected to particle size and FTIR analysis as previously mentioned in the methodology. The bacteria interaction and adhesion of the inoculants on HAp nanorod-assembled submicron particles have two distinct effects. According to Kerketta et al. (2025), Long and Wasaki (2023), and Yang and Yang (2020), *Enterobacter chuandaensis* and *Klebsiella variicola* is capable of Phosphate solubilization on the HAp nanorod-assembled submicron particles. This is confirmed by the FTIR spectra of decreased intensities of characteristic of the HAp nanorod-assembled submicron particles (Supplementary Figure 5). It is assumed that the solubilized $PO_{4}^{3-}$ is released into the CA matrix. The theoretical specific surface area can be estimated using the particle size analysis data. According to Doostmohammadi et al. (2011), the theoretical specific surface area can be calculated as follows using Equation 20


$$\begin{array}{c} D=\frac{6}{S_{sp}\rho_{a}} \end{array}$$ (23)

Where *D* is the hydrodynamic diameter; *S*_*sp*_ is the theoretical specific surface area; ρ_*a*_is the theoretical density of the material. The theoretical specific surface area of the HAp nanorod-assembled submicron particles has been reduced from 3.616*m*^2^*g*^−1^ to 3.341 *m*^2^*g*^−1^ indicating a decrease in the theoretical surface area of HAp nanorod-assembled submicron particles. According to Nuvoli et al. (2024), Tang et al. (2022) Yang et al. (2022), Yue et al. (2024), and Zhao et al. (2023), the negative surface charge of the bacterial cells adsorbs the *Ca*^2+^ in the CA beads and combines it with the released $PO_{4}^{3-}$ causes a microbe-induced precipitation on the cell surfaces in the form of *Ca*_5_(*P*_*O*_4_)3_*OH*. This causes an increase in the particle size of HAp nanorod-assembled submicron particles from 525 nm to 568.3 nm and the above-mentioned decrease in specific surface area. The proposed mechanism of cell adhesion and bacterial interactions with the HAp nanorod-assembled submicron particles is given in Supplementary Figure 5.

Two types of particles were observed after incubation with the bacterial inoculants. The larger particle size is due to the (568.3 nm) colonized HAp nanorod-assembled sub-micron particles and the smaller particles had a particle size of 125.6 nm (Figure 5f). The presence of the smaller HAp nanorod-assembled submicron particles can be due to two distinct reasons. According to Zhang et al. (2009), the selective adsorption of *Cit*^3−^ ions to the HAp nanorod-assembled submicron particle facets causes the growth orientation of nanorods of similar size (125.6 nm). Other reason is that according to Nuvoli et al. (2024), Tang et al. (2022) Yang et al. (2022), Yue et al. (2024), and Zhao et al. (2023), the bacteria-induced HA precipitation on the cell surface can get detached and dispersed in the medium which may cause the detection of smaller near-nano range HAp nanorod particles.

### 4.3 Biodegradability and biocompatibility of individual biocapsule components

According to Leather and Einhellig (1988), ψ has been the most utilized index and the most suitable to accurately describe germinability. λ is proven to be a more sensitive indicator for germinability and it is harder to measure compared to ψ. *GI* is an indicator which unites both λ and ψ. Higher *GI* is an indication of rapid seed germination (Wang et al., 2004). A high ψ values (< 80%) indicates non-phytotoxicity of the capsule and its individual counterparts (Bargougui et al., 2019). ψ for the biocapsule reached beyond 75% in all concentrations (Figure 12a). According to Courtney and Mullen (2008), the λ≥ 75% is considered to be a reliable indicator of non-phytotoxicity (Figure 12b). The phytotoxicity index was observed at a low level indicating a non-phytotoxicity to plants due to the biocapsule degradation (Figure 12d) (Enaime et al., 2020). Nevertheless, the *GI* was indicated more than 80%, only in 20% digestate concentration (Figure 12c). Even though λ and ψ are indicative of non-phytotoxicity, the overall *GI* across 50%0.75% and 90% digestate concentrations are below 80%. This might be due to the CMC breakdown of the biocapsule into smaller molecules such as low molecular weight organic acids and short-chain fatty acids, which can be phototoxic in high digestate concentrations. Furthermore, the localized oxygen depletion around roots of *Mung* seedlings caused by CMC degradation might cause GI to be lower than 80% (Kaur et al., 2023).

### 4.4 Composting analysis

#### 4.4.1 Intact biocapsule treatment enhances nutrient concentration, stabilization and thermogenesis

The *TN*_*dm*_% is subjected to gradual increase in all treatments, which agrees with previous studies of composting (Jusoh et al., 2013; Zhan et al., 2021). This can be attributed to the faster organic C biodegradation than N loss, which increases the N content per unit mass. As a result of *TN*_*dm*_% reduction, the *CN* ratio decreased during composting. According to Pepe et al. (2013), Qiu et al. (2020), and Zhao et al. (2023), the controlled dispersion of HuA in CA beads into the composting substrate can cause upregulation of denitrification genes of the native bacteria may increase the *TN*_*dm*_%. Furthermore, the increase in *TN*_*dm*_% during composting of $T_{biocapsule}^{S}$ can be attributed to the organic C loss in the form of CO_2_ during composting. Furthermore, the latter increase from 45d to 60d in *TN*_*dm*_% might occur due to the N–fixation occurring at the latter phase of composting. A decline in *TN*_*dm*_% can occur due to NH_3_ and ${\text{NO}}_{3}^{-}$-N volatilization, however, all the treatments were covered with plastic to retain moisture, which could lead to *TN*_*dm*_% preservation. Another approach to theorize the latter increase in *TN*_*dm*_% is due to the N–fixation that might occur due to the action of *E. chuandaensis* and *K. variicola* that might be capable of N-fixation in addition to lignin and cellulose degradation (Yang et al., 2018). This has to be examined further in future research. Nonetheless, this effect of latter N-improvement in seen in *T*_*control*_ also. But the difference was not statistically significant among the treatments (*p*> 0.01).

One of the important observations was that the rate of *TK*_*dm*_% increase was less in $T_{biocapsule}^{PS}$ compared to *T*_*control*_. This might be due to the premature release of the lignocellulolytic inoculant in $T_{biocapsule}^{PS}$ where the strains did not have enough time for proper sustained K diffusion and adapting to the microenvironment in compost feedstock. According to Iyengar and Bhave (2005), K is an element that is easily leached out. Nonetheless, the *TK*_*dm*_% increased in all the treatments. This can be attributed to the moisture retention and ability to maintain structural integrity and increase in porosity of RS during degradation avoiding the loss of K. This can be further backed by SEM observation on RS degradation under the influence of the lignocellulolytic inoculum used in $T_{biocapsule}^{S}$ and $T_{biocapsule}^{PS}$ treatments, where clear porous structures can be seen in degraded RS (Senadheera et al., 2025). Furthermore, the upward trend of *TK*_*dm*_% might also occur due to elevated microbial activity in RS composting. The mineral element concentration including *TK*_*dm*_% increased with a subsequent loss in dry mass of the feedstock.

According to Bernal et al. (2009), the overall increase in *TP*_*dm*_% all three treatments is attributed to elevated microbial activity during rice straw composting. The mineral element concentration increased with the subsequent loss of dry weight of composting feedstock. Nevertheless, incorporating organic N sources such as RS might increase the total nutrient content during composting (Xu Z. et al., 2006). Several studies suggest that RS incorporation can increase P availability (Sun et al., 2018). The rapid increase in *TP*_*dm*_% in $T_{biocapsule}^{S}$ and $T_{biocapsule}^{PS}$ compared to *T*_*control*_ can be theorized depending on studies by Schutter and Dick (2001) and Thevenot et al. (2010). The enhanced release of lignin from RS due to the action of ligninolytic *K. variicola* in $T_{biocapsule}^{S}$ and $T_{biocapsule}^{PS}$ might increase the *TP*_*dm*_% compared to *T*_*control*_. Nevertheless, the alkaline phosphomonoesterase activity might not be upregulated by the increase in the lignin content during composting. Therefore, the increase in the *TP*_*dm*_% might not be due to soil chemical processes rather than soil microbial processes. The increase in short and medium length cellulose chains due to the action of cellulolytic *E. chuandaensis* might facilitate the *TP*_*dm*_% fractions conversion from non-labile P pool in feedstock to the labile P pool via microbial assimilation (Zhou et al., 2015). One of the important factors to consider is that despite the high availability of lignin chains due to action of *K. variicola*, lignin does not promote alkaline phosphomonoesterase activity. Therefore, the availability of extra C source such as cellulose due to the cellulolytic action of *E. chuandaensis* might promote organic P mineralization via stimulation of synthesis of microbial phosphomonoesterases (Sun et al., 2018).

The overall loss of *TOC*_*dm*_% during composting is supported by previous studies (Rashad et al., 2010). The reduction of *TOC*_*dm*_% during rice straw composting is consistent with previous studies which used microbial inoculums to enhance *TOC*_*dm*_% degradation (Tran et al., 2015). However, studies using encapsulated lignocellulosic inoculums in rice straw composting are hardly found. The high rate of *TOC*_*dm*_%, reduction from 10d to 25d in $T_{biocapsule}^{S}$ can be attributed to the rapid degradation of crude fat, carbohydrates and proteins which are easily degradable coupled with faster lignin and cellulose degradation by well-established bacterial action caused due to sustained and slow release of the bacteria into the feedstock in $T_{biocapsule}^{S}$ treatment (Zhou et al., 2023).

According to Ravindran et al. (2018), *CN* ratio lower than 20:1 is considered as mature compost. Rapid decline in *TOC*_*dm*_% and *MC*_*dm*_% has an concerted effect on *CN* ratio decline (Gou et al., 2017). Even though *CN* ratio is considered as an important parameter for compost maturity, some authors argue it should not be used as a maturity index as different organic matter in waste leads to highly diverse *CN* ratio (Yuan et al., 2015). Therefore, it is suitable to use a higher initial *CN* ratio (~30:1) for composting of lignocellulosic agricultural waste such as RS than a lower *CN* ratio (~15:1) (Zhan et al., 2021).

K is immobilized in the phytoliths and the xylem tissue of rice straw (Zhai et al., 2023). According to Nguyen et al. (2014), the transported ionic K is trapped inside the phytoliths along with Si build up during the growth of the paddy leaves. During decalcification of the phytoliths the formation of microscopic hollow structures due to organic matter dissolution might release the trapped K into the microenvironment. A detailed analysis of the mechanism of rice straw degradation by the two isolates used in the biocapsule was done previously (Senadheera et al., 2025). During the study a division-of-labor mechanism where *K. variicola* AKL1104 degrades the surface lignin barrier initially and the cellulolytic *E. chuandaensis* AKC1108 causes secondary degradation of the exposed xylem and phloem tissue. During the coordinated rice straw degradation, SEM analysis of degraded rice straw surface revealed porous morphology similar to the effect described by Nguyen et al. (2014). This effect can be attributed to the marked increase in *TK*_*dm*_% during 25–35d composting in biocapsule treatments. Furthermore, the *K. variicola* and *E. chuandaensis* are well-established K solubilizes on lignocellulosic substrates (Zhang and Kong, 2014; Bao et al., 2025; Zhai et al., 2023). The secretion of organic acids by both bacterial species will have more accessibility of immobilized K due to the porous effect caused by the synergistic rice straw degradation.

The accelerated increase of *TN*_*dm*_%, *TP*_*dm*_%, and *TK*_*dm*_%, in the biocapsule treatments where *K. variicola* and *E. chuandaensis* is with consistent with the reports by Dhanda and Saharan (2025). During the study Dhanda and Saharan (2025), used *K. variicola* and *E. chuandaensis* as microbial inoculants during composting. According to Shukla et al. (2016) and Barrena et al. (2007), increase of dehydrogenase activity of *K. variicola* and *E. chuandaensis* causes a marked increase of dehydrogenase activity during first 30d of composting. Furthermore, elevated secretion of alkaline phosphatase by *K. variicola* and *E. chuandaensis* causes rapid solubilization of P which in return causes a marked increase in *TP*_*dm*_% during first 20d composting (Li et al., 2018; Xu D. et al., 2006; Li et al., 2020; Raut et al., 2008).

Even though, the rice straw degradation in the compost pile is done synergistically by the dual-strain inoculants, *E. chuandaensis* population in the early days of composting (25–35d) might cause denitrification by converting $NO_{3}^{-}-N$ into *N*_2_ by utilizing the metabolic intermediates of cellulose degradation due to its cellulolytic ability, which in return can cause reduction of *TN*_*dm*_%. However, the presence of ligninolytic bacterial species such as *K. variicola* inhibits the dentification caused by the early stages of composting via denitrification inhibition and allows a marked increase in *TN*_*dm*_% and $NO_{3}^{-}-N$ in early stages of composting (Mei et al., 2022; Liu et al., 2020; Wang et al., 2021). As a bottom-line, the exposure of the inner xylem and phloem tissues due to the sequential degradation of the dual consortium, the exposure of the cellulose, xylose and hemicellulose alongside the proteinaceous compounds in the inner paddy straw tissues allows a rapid metabolic turnover of the immobilized N, P, and K due to the biodegradative action of the consortium as well as the native microbiota.

#### 4.4.2 Intact biocapsule treatment accelerates composting dynamics through enhanced lignocellulolysis

The rapid rise in the pH from 15d to 20d is due to the ammonification in all the treatments. Nonetheless, from 5d to 20d, the increase in pH $T_{biocapsule}^{S}$ and $T_{biocapsule}^{PS}$ is higher compared to *T*_*control*_. This can be attributed to the encapsulation in $T_{biocapsule}^{S}$ and $T_{biocapsule}^{PS}$ provide controlled bacterial release and leads to early lignocellulolysis, faster ammonia conversion (due to decomposition of amino acids) and rapid humification (Ravindran et al., 2018). Nonetheless, the high bio-degradation and of the intact bio-capsule components assists the establishment of the inoculants in the composting feedstock. The bio degradation products of the bio capsule components have no detrimental ecological influence (Figures 14a–f). The $T_{biocapsule}^{S}$ reaches the pH neutrality faster than $T_{biocapsule}^{PS}$ and *T*_*control*_ (Figure 13a). The delayed pH neutrality in *T*_*control*_ signals delayed humification and N stabilization (Figure 14a). Even though, $T_{biocapsule}^{PS}$ leads to pH normalization early than *T*_*control*_, the rapid decline in microbial viability due to ammonia stress which is caused due to the powdered state of the biocapsule may hinder the pH neutralization in compost. The gradual release of the bacterial inoculants in $T_{biocapsule}^{S}$ may allow the compost piles to gain pH neutrality quicker than $T_{biocapsule}^{PS}$ and *T*_*control*_. The results are consistent with studies by Meng et al. (2018).

The rapid rise of EC from 10 to 20 days in $T_{biocapsule}^{S},\;T_{biocapsule}^{PS}$ and *T*_*control*_ can be attributed to the ammonification and decomposition or organic matter. None of the treatments reached the maximum EC (3.0 *DS ml*^−1^) threshold for phytotoxicity during composting (Ravindran et al., 2018). The EC level of the compost reached acceptable levels (< 1.5 *DS ml*^−1^) in 30 and 35 days for $T_{biocapsule}^{S}$ and $T_{biocapsule}^{PS}$ and reached a final EC of 0.62 ± 0.00 *DS ml*^−1^ and 0.74 ± 0.01 *DS ml*^−1^ for $T_{biocapsule}^{S}$ and $T_{biocapsule}^{PS},$ respectively, which can be assumed that the compost is non-phytotoxic in nature (Li et al., 2019). The reduction in EC in all three treatments was caused by ammonia evaporation, mineral salt precipitation and reduction in water-soluble components in compost (Rashad et al., 2010). One-way ANOVA analysis showed a significant difference among the treatments (*p* < 0.05).

The initial *MC*_*dm*_% was maintained at 60% to prevent the nutrient washout and reduction of air volume and ultimately prevent microbial activity deterioration. It was essential to maintain the *MC*_*dm*_% more than 50% to initiate decomposition in all three treatments. The higher degree of moisture loss in $T_{biocapsule}^{S}$ and $T_{biocapsule}^{PS}$ compared to *T*_*control*_ is due to high moisture consumption during selective lignin and cellulose decomposition in RS by concerted action of added inoculants and indigenous microbes in $T_{biocapsule}^{S}$ and $T_{biocapsule}^{PS}$ (Asina et al., 2016; Du et al., 2021).

All the treatments showed the four distinct phases (heating phase; ambient to~ 40 °C, high-temperature phase/thermophilic phase; ~40–65 °C), cooling phase; ~65 °C ~40 °C) and mature phase; ~20–30 °C) of composting process (Cornell Waste Management Institute, 2025). At the end of 60d, all the three treatments did not reach the room temperature. This implies that it will make more time (greater than the timer period of 60 d) for the compost to reach completely mature phase with stable temperatures. According to Jusoh et al. (2013), the use of RS has a considerable effect on temperature increase during the early stages of composting. The high *CN* ratio of RS gives room for the microbes for heat generation in the process of organic matter decomposition. However, the rate of temperature increases in $T_{biocapsule}^{S}$ and $T_{biocapsule}^{PS}$ is higher compared to *T*_*control*_. This can be attributed to the concerted lignocellulolysis by the two inoculants in $T_{biocapsule}^{S}$ and $T_{biocapsule}^{PS}$. The thermophilic phase of composting should exceed 55 °C for more than 15d to eliminate harmful pathogens and weeds in the feedstock and obtain contaminants-free final product. $T_{biocapsule}^{S}$ and $T_{biocapsule}^{PS}$ reached the high thermophilic temperatures compared to *T*_*control*_. This can be attributed to the labile C in Cow dung and its thermal inertia (Bustamante et al., 2008). It is assumed that the sand might have been introduced during the sampling, feedstock mixing.

According to Lasaridi et al. (2005), Manipura et al. (2025), and Sewmi et al. (2025), the competition due to increased demand in Sri Lanka for compost fertilizer has caused an emergence of low quality commercial products entering the market and ultimately lose the trust of farmers. Therefore, the SLS has applied a minimum statutory minimum amount for *TOC*_*dm*_%to be above 20%, to prevent the misleading of products with blending of materials such as soil offered as composts to customers.

Nonetheless, the *TOC*_*dm*_% for $T_{biocapsule}^{S}$ is 18.48 ± 0.16% which is lower than the preferred standard of SLS. According to Roca-Pérez et al. (2009) and Zhan et al. (2021), *TOC*_*dm*_% of ~18% indicates the sufficient and stable humification of the organic matter in the compost where a majority of the organic matter has been concerted to humic substances. Both $T_{biocapsule}^{S}$ and $T_{biocapsule}^{PS}$ treatments achieved *TOC*_*dm*_% near 18% which signifies the stable state of the compost produced. The *TOC*_*dm*_% of $T_{biocapsule}^{S}$ is lower than $T_{biocapsule}^{PS}$ treatment (Jurado et al., 2015). This can be attributed to the fact that the intact biocapsule allows the native bacteria and the entrapped inoculants to colonize the biocapsule and degrade the capsule layers while releasing easily metabolisable small molecules such as monosaccharides and disaccharides which can proliferate the bacterial cell density in high concentrations in a localized area (where the biocapsule is buried) and spread to the rest of the regions of the compost pile rapidly. The powdered biocapsule has a sporadic positioning of the biocapsule components in the compost pile and the amount of the polymeric substances is low in each location for the native microbes and entrapped inoculant to colonize and degrade the polymers to proliferate to a great number, which in return will cause the biodegradation of organic matter (Firmanda et al., 2022).

The significant differences in nutrient metrics and physical quality parameters between $T_{biocapsule}^{S}$ and $T_{biocapsule}^{PS}$ as depicted in Table 5, shows that the MRH and RSOCS plays a significant role in sustained release of the inoculum, offers protected microenvironment with nutrient and moisture availability for the inoculants to better adapt to the lignocellulosic substrate for degradation. The effect of RSOCS and MRH of inoculant colonization, succession and lignocellulosic degradation is investigated in the next phase of the research as a continuation.

## 5 Conclusion

The intact biocapsule and its components showed good biodegradability and biocompatibility making it suitable as a platform for future microbial agent delivery systems for agricultural purposes. The bacterial encapsulation and cell release from the system allowed a rapid but controlled release of the inoculum. The intact biocapsule consistently demonstrated greater composting enhancement potential across composting parameters. The $T_{biocapsule}^{S}$ treatment demonstrated pH stabilization, greater moisture loss and efficient organic matter mineralization along with improved concentrations of N, P and K. Nevertheless, all the treatments, including control treatment reached the desired composting standards of Sri Lankan Standards (SLS) 1636: 2019 for compost made from raw materials of agricultural and animal origin. In contrast, $T_{biocapsule}^{PS}$ treatment with powdered biocapsule showed reduced efficiency compared to $T_{biocapsule}^{S}$. Premature microbial release, lack of structural protection might reduce the composting efficiency of the inoculum in $T_{biocapsule}^{PS}$. The utilization of lignocellulolytic inoculants and the mode of delivery using biocapsule had an effect on thermogenesis and heat retention during composting where the controlled release of microbes by $T_{biocapsule}^{S}$ being the most successful. The mechanism of microbial delivery during the decomposition of the biocapsule in composting feedstock should be further investigated to further improve its effectiveness and will be investigated in future research using the biocapsule.

## Data Availability

The original contributions presented in the study are included in the article/Supplementary material, further inquiries can be directed to the corresponding author.
