# Supplementary material for: A novel solid-state, multi-layered biodegradable microbial inoculant system for rice straw composting: biocapsule design, characterization, and performance evaluation
Source: Front Microbiol. 2026 Apr 17;17:1758888. doi: 10.3389/fmicb.2026.1758888 (PMC13134392; doi:10.3389/fmicb.2026.1758888)
Supplement: Supplementary file 1 [file Data_Sheet_1.pdf]

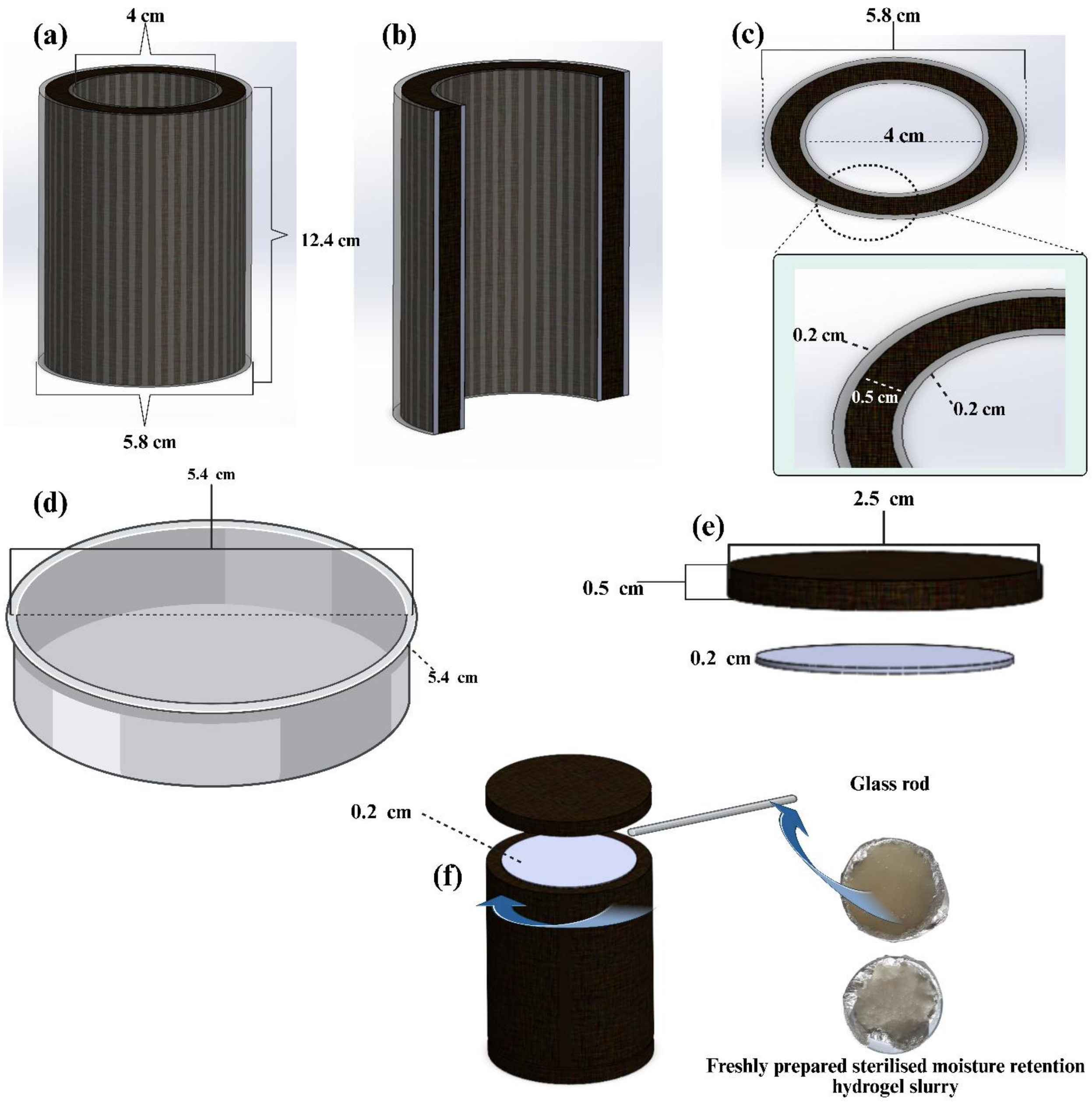

Figure 1 – Standardization of the biocapsule assembly with RCOCS filled inside (brown colored). (a) dimensions of the aluminium mold; (b) cross-section of the aluminium mold with RCOCS filled inside (brown colored); (c) Birds-eye view of the aluminium mold with RCOCS inside; (d) aluminium mold of the RCOCS lid; (e) Side-view of the RCOCS lid; (f) fitting of the RCOCS lid to the rest of the RCOCS cylinder.

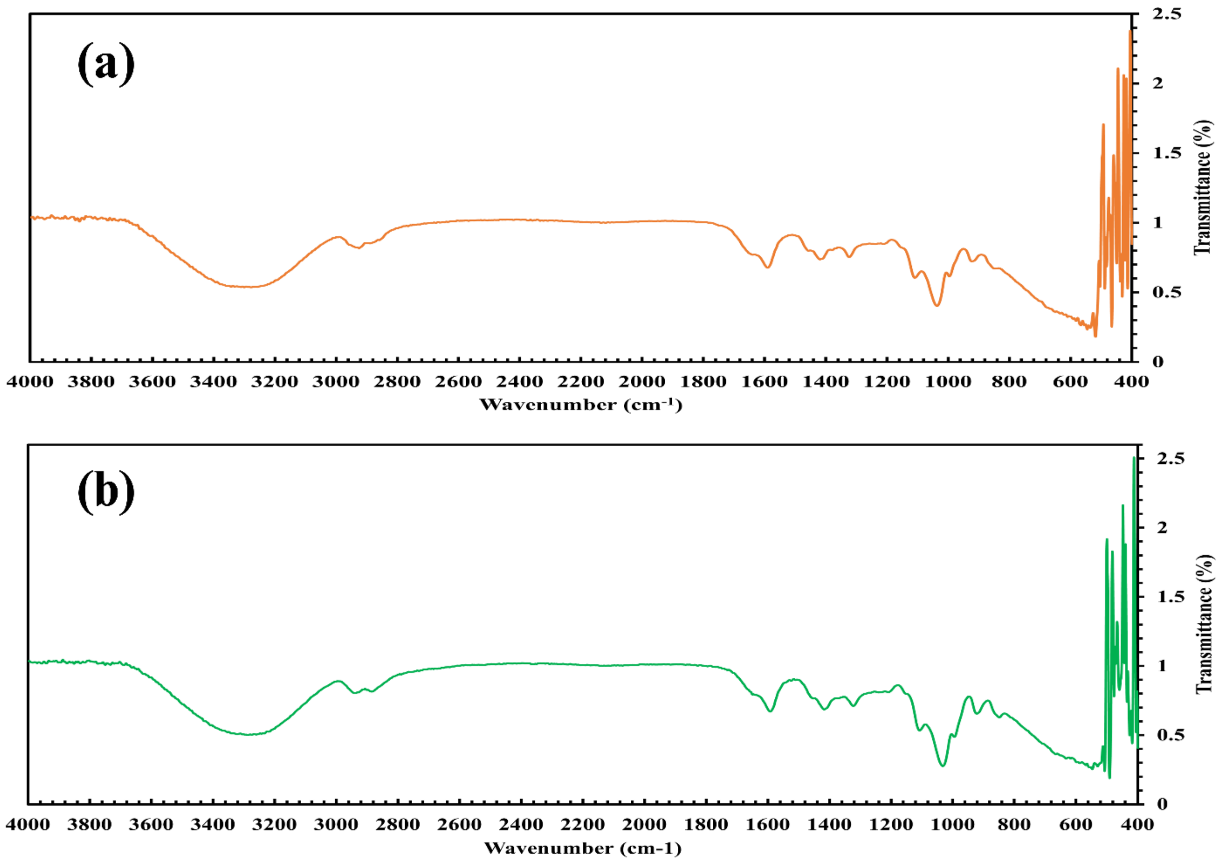

Figure 2 – FTIR of rice straw derived – humic acid; (b) FTIR spectra of commercial humic acid.

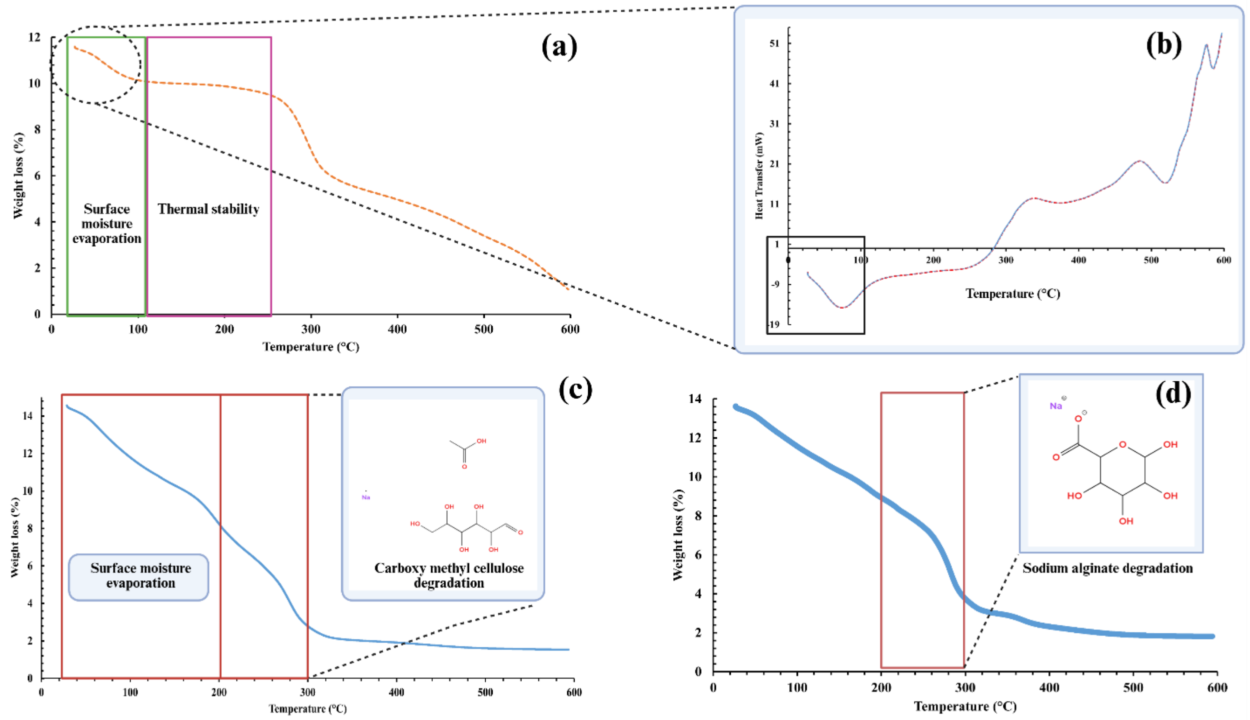

Figure 3 – (a) Thermogravimetric analysis of outer rice straw reinforced biocapsule composite shell; (b) differential calorimetric thermal flow across the outer rice straw reinforced biocapsule composite shell; (c) thermogravimetric analysis of moisture retention hydrogel; (d) thermogravimetric analysis of calcium alginate gel beads.

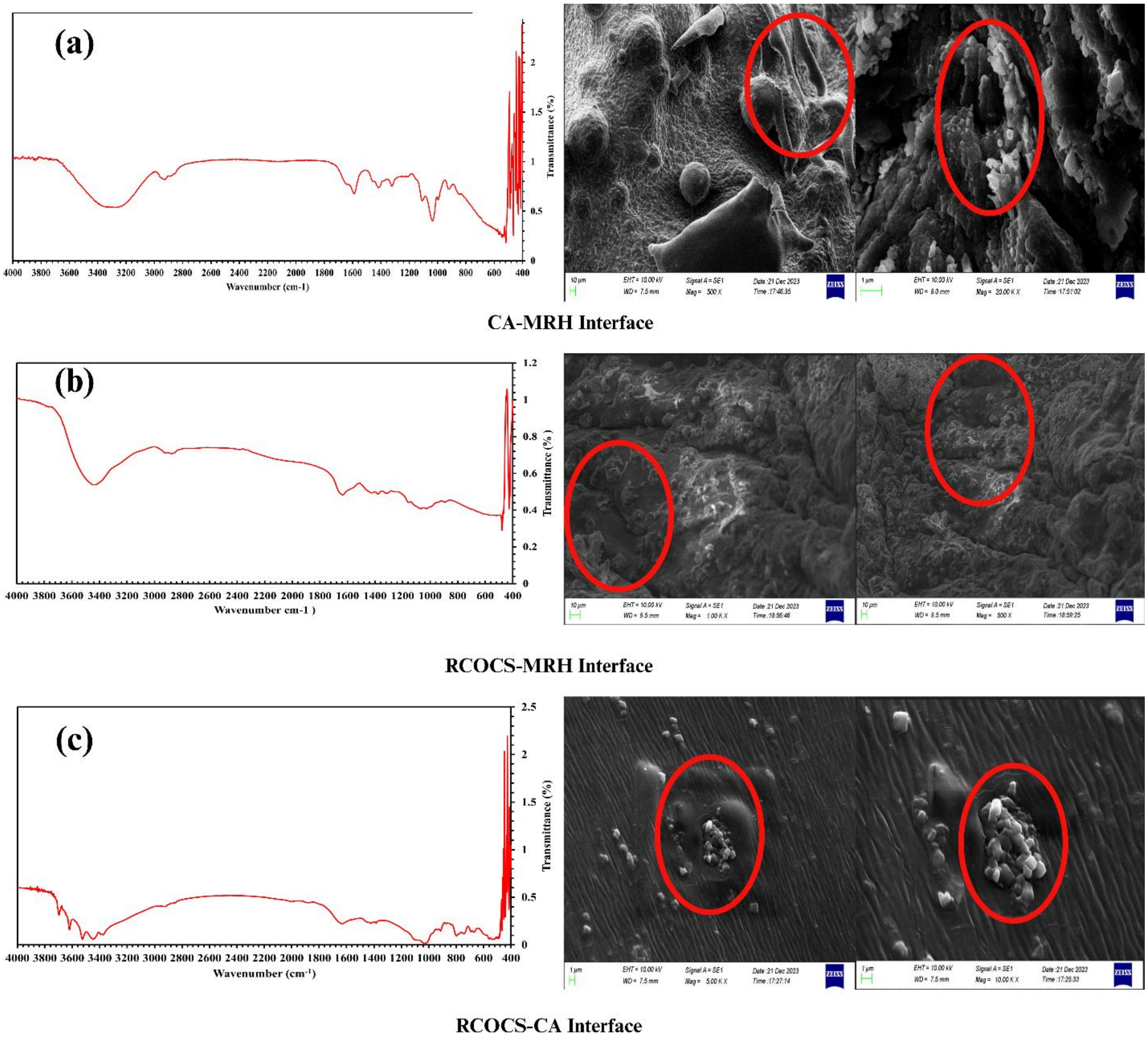

Figure 4 – (a) Calcium alginate- moisture retention hydrogel interface (FTIR on the left, SEM image on the right); (b) outer rice straw reinforced biocapsule composite shell-moisture retention hydrogel (FTIR on the left, SEM image on the right); (c) outer rice straw reinforced biocapsule composite shell-calcium alginate interface (FTIR on the left, SEM image on the right)

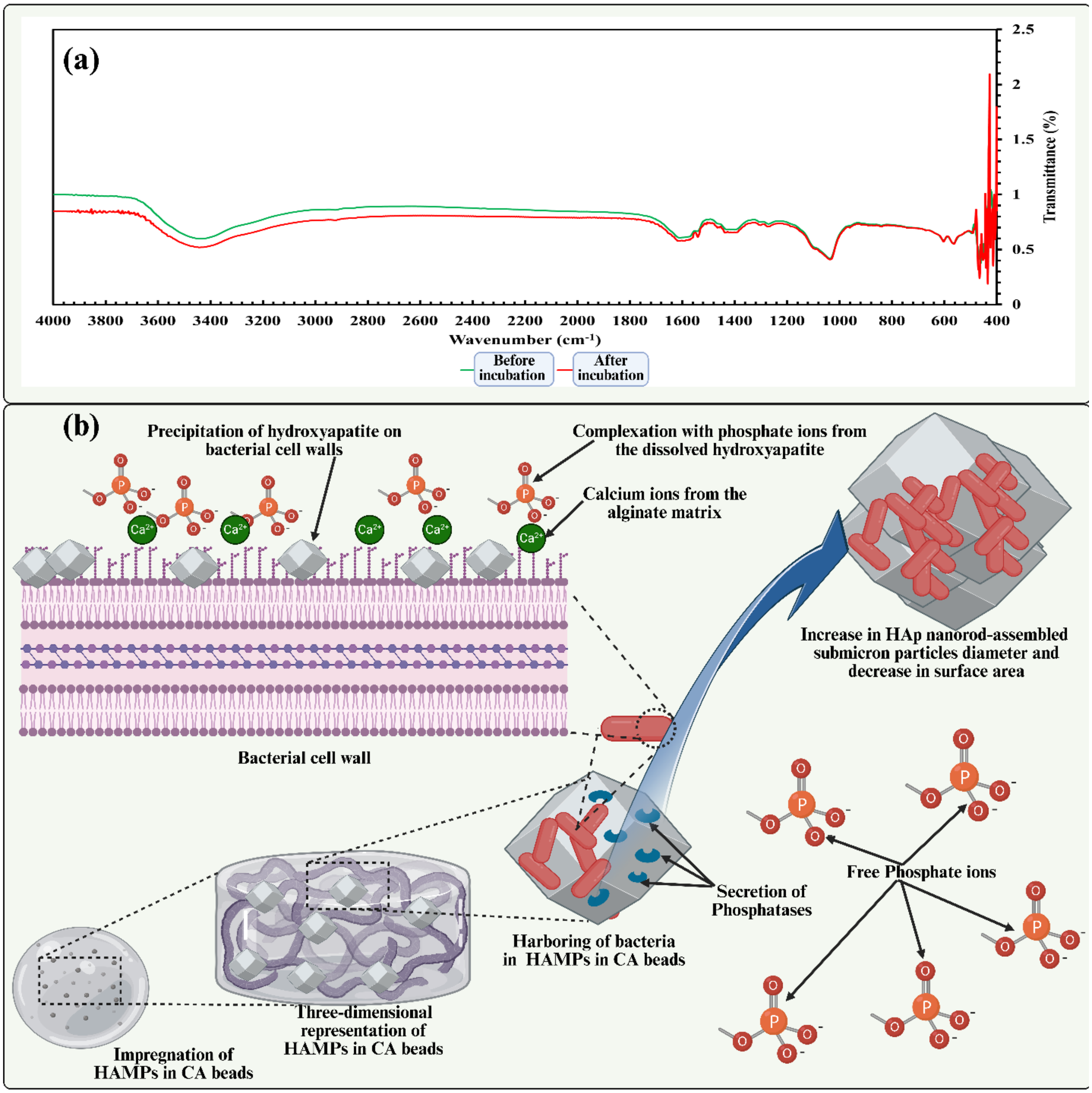

Figure 18 – (a) Direct comparison of FTIR spectra of HAp nanorod-assembled submicron particles without bacterial adhesion and with bacterial adhesion; (b) proposed mechanism of bacterial cell release and the bacterial-cell wall induced HAp precipitation.
